# Structural assemblies of the di- and oligomeric G-protein coupled receptor TGR5 in live cells: an MFIS-FRET and integrative modelling study

**DOI:** 10.1038/srep36792

**Published:** 2016-11-11

**Authors:** Annemarie Greife, Suren Felekyan, Qijun Ma, Christoph G. W. Gertzen, Lina Spomer, Mykola Dimura, Thomas O. Peulen, Christina Wöhler, Dieter Häussinger, Holger Gohlke, Verena Keitel, Claus A. M. Seidel

**Affiliations:** 1 Chair for Molecular Physical Chemistry, Heinrich Heine University Düsseldorf, 40225 Düsseldorf, Germany; 2 Institute for Pharmaceutical and Medicinal Chemistry, Heinrich Heine University Düsseldorf, 40225 Düsseldorf, Germany; 3 Clinic for Gastroenterology, Hepatology and Infectious Diseases, Heinrich Heine University Düsseldorf, 40225 Düsseldorf, Germany

## Abstract

TGR5 is the first identified bile acid-sensing G-protein coupled receptor, which has emerged as a potential therapeutic target for metabolic disorders. So far, structural and multimerization properties are largely unknown for TGR5. We used a combined strategy applying cellular biology, Multiparameter Image Fluorescence Spectroscopy (MFIS) for quantitative FRET analysis, and integrative modelling to obtain structural information about dimerization and higher-order oligomerization assemblies of TGR5 wildtype (wt) and Y111 variants fused to fluorescent proteins. Residue 111 is located in transmembrane helix 3 within the highly conserved ERY motif. Co-immunoprecipitation and MFIS-FRET measurements with gradually increasing acceptor to donor concentrations showed that TGR5 wt forms higher-order oligomers, a process disrupted in TGR5 Y111A variants. From the concentration dependence of the MFIS-FRET data we conclude that higher-order oligomers – likely with a tetramer organization - are formed from dimers, the smallest unit suggested for TGR5 Y111A variants. Higher-order oligomers likely have a linear arrangement with interaction sites involving transmembrane helix 1 and helix 8 as well as transmembrane helix 5. The latter interaction is suggested to be disrupted by the Y111A mutation. The proposed model of TGR5 oligomer assembly broadens our view of possible oligomer patterns and affinities of class A GPCRs.

TGR5 (GPBAR-1, M-BAR) is the first identified G-protein coupled bile acid receptor^1^ and is widely expressed in tissues, including liver, intestine, and the central and enteric nervous system^2^,^3^. Animal studies suggest that TGR5 activation leads to anti-inflammatory effects and influences energy homeostasis and glucose metabolism, thereby playing a role in the pathogenesis of obesity and diabetes^4^. Therefore, TGR5 has emerged as a potential therapeutic target to treat metabolic disorders. The most potent TGR5 bile acid agonist is taurolithocholic acid (TLCA/TLC)^1^. In model cell lines it was shown that TGR5 couples to Gα_s_, leading to stimulation of adenylate cyclase (AC) and formation of cyclic AMP (cAMP)^1^.

To date, no high-resolution crystal structure of TGR5 is available, and knowledge on TGR5 regulation and oligomerization is scarce. Homology models of TGR5 have been presented based on template structures of other seven transmembrane (7TM) domain receptors^5^,^6^,^7^,^8^. We previously reported that the amino acids 285–294 at the TGR5 C-terminus form an alpha-helical stretch important for plasma membrane localization and thus responsiveness to extracellular ligands^9^.

It is now well established that class C GPCRs form homo- and hetero-oligomers^10^. Oligomer formation of GPCRs affects a broad range of biological functions ranging from intracellular trafficking, protein turnover, receptor function, signal enhancement or blockage upon ligand binding, G-protein independent signaling to internalization and receptor desensitization (for an overview see refs 11 and 12). However, for class A GPCRs such as TGR5, there are controversial data about the functional significance of homo- and hetero-oligomer formation^10^. Studies with rhodopsin^13^,^14^, μ-opioid^15^ and β_2_-adrenergic receptors trapped as either monomers or dimers in nanodiscs demonstrated that monomers are functional and activate G-proteins; sometimes monomers are even more efficient than homo-dimers^10^. The same GPCRs were also shown to be stable as dimers or tetramers in living cells^13^,^14^,^15^. Many researchers proved at least dimerization by using biophysical approaches such as bioluminescence and Förster Resonance Energy Transfer techniques (BRET and FRET), as well as single molecule analysis^16^ and atomic force microscopy in native disc membranes^17^. FRET describes the distance dependent energy transfer from an excited donor (D) to an acceptor (A) fluorophore and is used to study biomolecules in living cells which are fused to genetically encoded fluorescent proteins (FP) for convenience, although other molecular tags are also being used.

Several oligomer models exist for GPCRs, based on predictions of relative stabilities of dimer interfaces by molecular simulations and bioinformatics studies as well as wet-lab techniques. Extended biased molecular dynamics simulations suggested a model in which homo-dimers characterized by stable interactions involving transmembrane helix 1 (TM1) transiently interact with the other protomer via other helices such as TM4^18^. Bioinformatics studies predicted a role for transmembrane helices TM1 and TM4 to TM6 in dimerization; mutation of residues in this region disrupted dimerization^19^,^20^. AFM, crystallography and FRET studies of the β_1_- and β_2_-adrenergic receptors^21^, muscarinic receptor M_3_^22^, rhodopsin^17^,^23^ and the μ-opioid receptor^24^ suggested that oligomerization interfaces are most probably formed by TM1-TM2-helix(H)8 and TM4-TM5 or TM5-TM6. So far, several spatial arrangements of tetrameric GPCRs are discussed. For muscarinic receptor M_3_ a rhombic arrangement of tetramers seems to be preferred rather than linear or squared ones^22^, whereas for rhodopsin either a more linear or squared arrangement are discussed^10^,^17^,^22^. We will discuss our data in respect to these findings to suggest TGR5 oligomerization models.

To perform protein-protein interaction studies in living cells without disturbance and with high spatial resolution, we applied Multiparameter Image Fluorescence Spectroscopy (MFIS). It combines fluorescence lifetime imaging and fluorescence anisotropy microscopy allowing a comprehensive analysis of the biophysical properties of homo- and heteromeric molecular complexes by FRET. MFIS is based on Multiparameter Fluorescence Detection (MFD), which has been established as a standard tool to investigate biomolecules in *in vitro* experiments^25^,^26^,^27^. Similar to MFD, MFIS-FRET records photons one by one, which allows for parallel recording of all fluorescence parameters (fundamental anisotropy, fluorescence lifetime, fluorescence intensity, time, excitation spectrum, fluorescence spectrum, fluorescence quantum yield, and distance between fluorophores) and additionally pixel/image information over time periods of hours with picosecond accuracy. The multidimensional analysis of correlated changes of several parameters measured by FRET, fluorescence fluctuation, fluorescence lifetime and anisotropy increases the robustness of the analysis significantly. The economic use of photon information even allows detection of fluorescent fusion proteins that are expressed at very low levels. We already showed the reliability of this technique for molecular interaction studies in different environments in human and plant cells^28^,^29^.

The main focus of this study is to use a combined strategy applying cellular biology, co-immunoprecipitation experiments, MFIS-FRET, molecular modelling and simulations to obtain information about oligomerization of TGR5 and the influence of a mutation in the TGR5 ERY domain on oligomerization.

## Results

### TGR5 forms homo-complexes but the complex affinity differs between TGR5 variants

To characterize the complex formation of TGR5, we used three TGR5 variants, TGR5 wt, TGR5 Y111A, and TGR5 Y111F. The tyrosine residue at position 111 is part of the highly conserved ERY motif, which is important for GPCR function^30^ and also predicted to be phosphorylated by EGFR using NetPhos^31^.

**Figure 1:**
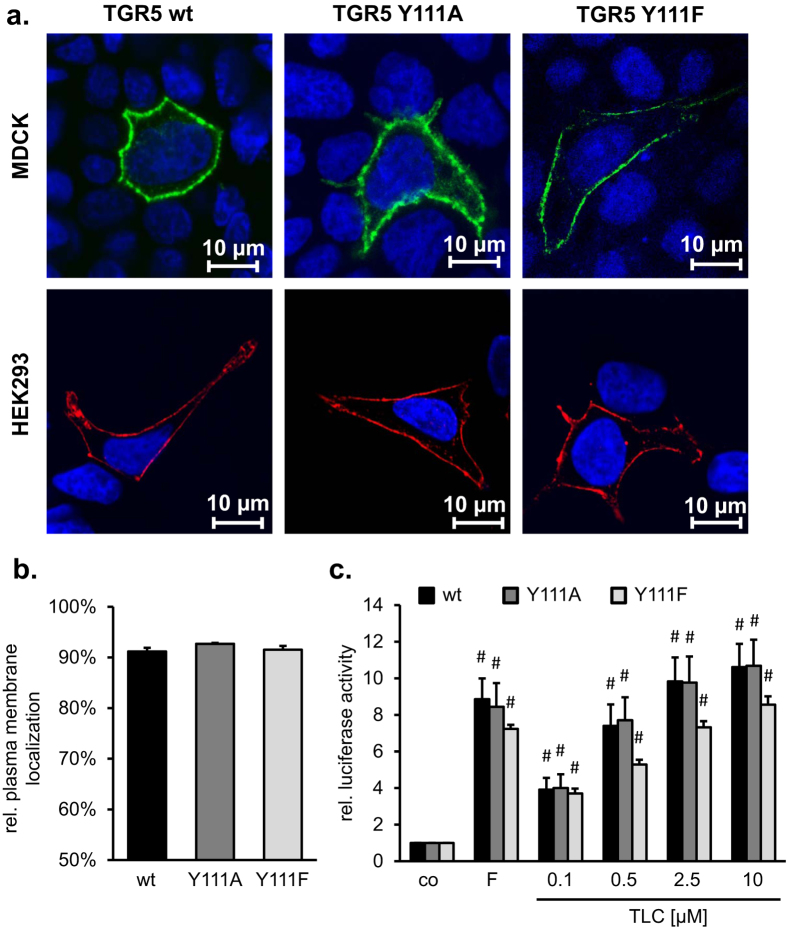
Localization and functional analysis of TGR5 wt and Y111 variants. (**a**) Localization of TGR5 by confocal laser scanning microscopy. MDCK cells (upper panels) were transiently transfected with FLAG-TGR5-YFP constructs. The YFP-fluorescence was detected in the plasma membrane for TGR5 wt as well as for the TGR5 Y111A and TGR5 Y111F variants. HEK293 cells (lower panels) were transiently transfected with TGR5-pcDNA constructs. TGR5 was stained using the RVLR2 antibody (in red). TGR5 as well as the TGR5 Y111A and TGR5 Y111F variants were present in the plasma membrane. Nuclei were stained with Hoechst (blue). Bars = 10 μm. (**b**) Relative quantification of TGR5 plasma membrane localization using flow cytometry. The amount of FLAG-TGR5-YFP within the plasma membrane corresponds to the amount of positive FLAG-tag labelling (=extracellular labelling) divided by the total amount of YFP-fluorescence. TGR5 Y111A and TGR5 Y111F were detected on the cell surface in 92.7% and 91.5% of the transfected cells, which was similar to the TGR5 wt with 91.2% (n = 3 independent transfection experiments). (**c**) TGR5 receptor activity was determined using a cAMP responsive luciferase assay. HEK293 cells were co-transfected with TGR5 (pcDNA3.1+), a cAMP responsive luciferase reporter construct, and a Renilla expression vector. Luciferase activity served as a measure of the rise in intracellular cAMP following activation of TGR5. Forskolin (F, 10 μM) was used as TGR5-independent positive control. TGR5 Y111A and TGR5 Y111F did not affect receptor responsiveness to the bile acid taurolithocholate (TLC). Results (wT n = 8; TGR5 Y111A n = 9; TGR5 Y111F n = 6) are expressed as mean + SEM. ^#^Significantly different (*p* ≤ 0.01) from DMSO (co = control).

Immunofluorescence staining in MDCK and HEK293 cells as well as FACS analysis of transfected HEK293 cells demonstrated that all TGR5 variants were correctly localized at the plasma membrane in about 92% of the transfected cells (Fig. 1a,b). Furthermore, TGR5 responsiveness towards TLC was investigated using a cAMP-responsive luciferase assay^9^, where luciferase activity served as a measure for the second messenger cAMP following TGR5 activation. Forskolin (F) elevates cAMP independent of TGR5 and was used as positive control. Stimulation of TGR5 wt, TGR5 Y111A, or TGR5 Y111F with TLC led to a significant dose-dependent increase in luciferase activity in all three cases (Fig. 1c).

**Figure 2:**
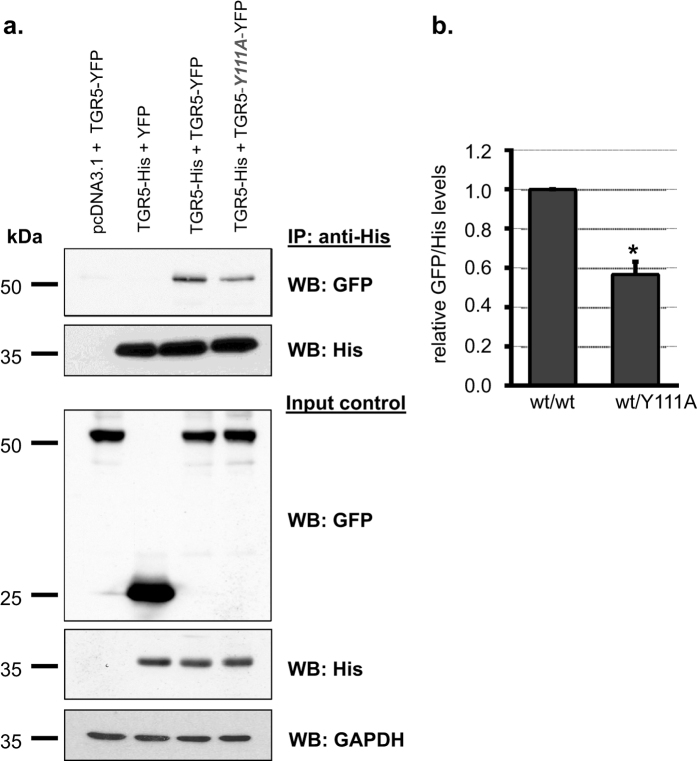
Detection of TGR5 multimerization by co-immunoprecipitation. (**a**) HEK293 cells were transiently transfected with pcDNA3.1 and TGR5-YFP, TGR5-His and pEYFP, TGR5-His and TGR5-YFP, or TGR5-His and TGR5 Y111A-YFP. Immunoprecipitation (IP) was carried out using an anti-His antibody. Equal volumes of the precipitate were deglycosylated with N-glycosidase-F, separated by SDS-PAGE, and blotted onto PVDF membranes. For Western blotting (WB) horseradish-peroxidase-coupled primary antibodies against His and GFP were used. TGR5-YFP was co-precipitated with TGR5-His. Mutation of tyrosine 111 to alanine in TGR5-YFP reduced the amount of co-precipitated variant receptor. Cell lysates (50 μg total protein lysates served as input controls and were separated by SDS-PAGE and proteins were blotted onto PVDF membranes. WB was carried out with horseradish-peroxidase-coupled primary antibodies against His and GFP or an antibody against glyceraldehyde-3-phosphate dehydrogenase (GAPDH). (**b**) Densitometric analysis of the anti-GFP and anti-His Western blots. Relative TGR5-TGR5 interaction was determined as relative GFP to His levels. Results are expressed as mean + SEM (n = 4), *Significantly different from wt-His/wt-YFP interaction, *p* < *0.05*.

To analyze the interaction between TGR5 wt proteins or TGR5 wt with TGR5 Y111A, we performed Co-immunoprecipitation experiments (Co-IP). His-tagged TGR5 wt and either TGR5 wt-YFP or TGR5 Y111A-YFP proteins were expressed in HEK293 cells. Immunoprecipitation of His-tagged TGR5 wt was carried out with an anti-His antibody. The interaction of TGR5 proteins was visualized using an anti-GFP antibody, which recognized the TGR5 C-terminal YFP (Fig. 2a lane 3). Co-IP clearly showed that TGR5 forms homo-complexes. Compared to the interaction between TGR5 wt proteins, the interaction between TGR5 wt and TGR5 Y111A is significantly reduced by about 40% as measured by densitometry (Fig. 2b).

### Pixel-wise MFIS-FRET analysis demonstrates remarkable differences in FRET properties between TGR5 variants

**Figure 3:**
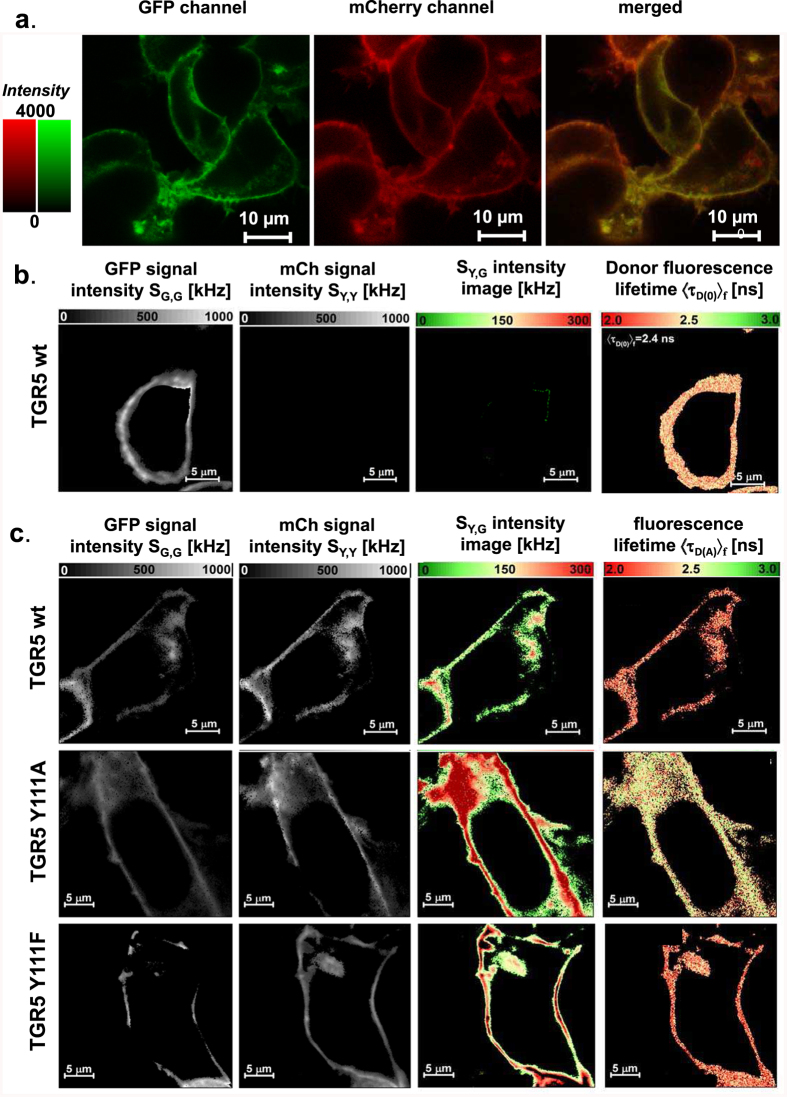
Detection of TGR5 multimerization by pixel-wise MFIS-FRET analysis. (**a**) HEK293 cells, transiently transfected with TGR5-GFP and TGR5-mCherry (transfection ratio 1:10), were imaged for co-localization of GFP and mCherry using sequential scanning and a scanning resolution of 1024 × 1024 pixels. Each TGR5-GFP and TGR5-mCherry picture is shown in a false color saturation mode and then overlaid by using green and yellow intensity colors. TGR5 wt-GFP and TGR5 wt-mCherry are clearly co-localized at the cell membrane. Scale bar 10 μm. The TGR5 Y111 variants are shown in SI Fig. 1. (**b**) MFIS analysis of TGR5 wt-GFP transfected HEK293 cells by comparing (from left to right) the signal intensity of the donor GFP (*S*_*G,G*_), signal intensity of the acceptor mCherry (*S*_*Y,Y*_), the detection of yellow mCherry photons after excitation of GFP (*S*_*Y,G*_, *S*: signal, *Y*: yellow emission, *G*: green excitation) as a result of FRET, and changes in the donor fluorescence lifetime 〈*τ*_*D*(*0*)_〉_*f*_. For TGR5 wt-GFP only the donor signal but no acceptor signal is detected. The MFIS analysis of TGR5 Y111 variants is shown in SI Fig. 1. (**c**) The same parameters were used for TGR5 GFP/mCherry samples. The MFIS measurements show FRET (*S*_*Y,G*_ and changes in 〈*τ*_*D*(*A*)_〉_*f*_) in all TGR5 variants, which indicates at least homo-dimerization.

To further analyze differences in the complex formation found by Co-IP we used the genetically encoded fluorescent proteins GFP and mCherry attached to the C-terminus of TGR5 to measure FRET by MFIS-FRET in live cells. GFP and mCherry are commonly used as a FRET pair with a Förster radius R_0_ = 52 Å^32^. As shown in Fig. 3a and SI Fig. 1a, all TGR5-GFP and TGR5-mCherry variants (wt, Y111A and Y111F) are strongly co-localized at the cell membrane of HEK293 cells. To visualize the heterogeneity within and between cells, the MFIS-FRET images were accurately analyzed in a pixel-wise manner to compute all relevant fluorescence parameters. During this procedure, photons are pixel-wise selected, grouped according to their properties, and selectively integrated to reduce noise (see SI methods). For a direct proof of FRET, it is necessary to show that the observed signal changes are due to differences in FRET efficiency *E* and not due to local changes of fluorophore properties or transfection artifacts. Thus, it is mandatory to analyze both FRET indicators: (i) FRET-induced donor quenching due to the presence of acceptor and (ii) the occurrence of FRET-sensitized acceptor fluorescence^33^.

A selection of these relevant FRET indicators is displayed in images of the TGR5 wt donor-only reference sample (Fig. 3b) and the FRET sample (Fig. 3c, first row): Signal intensity *S* of the donor GFP in the green detection channel by donor excitation (*S*_*em,ex*_ = *S*_*G,G*_; λ_ex_ = 488 nm), signal intensity of the directly excited acceptor mCherry in the yellow detection channel *S*_*Y,Y*_ (λ_ex_ = 559 nm), and as a result of FRET the FRET-sensitized mCherry signal *S*_*Y,G*_. Moreover, the quenching of the donor by FRET is judged by comparing the fluorescence-weighted average lifetimes of the donor in absence 〈*τ*_*D*(*0*)_〉_*f*_  and presence of acceptor 〈*τ*_*D*(*A*)_〉_*f*_, respectively. If no FRET occurs, we only expect signals in the green channel. This is indeed observed for the reference measurement TGR5-GFP (Fig. 3b). Furthermore, 〈*τ*_*D*(*0*)_〉_*f*_  does not change, as expected.

Compared to cells transfected with the donor-only reference TGR5-GFP (Fig. 3b), the MFIS-FRET measurements of the FRET sample suggest the presence of FRET, as the FRET-sensitized acceptor signal was detectable (Fig. 3c, S_Y,G_ image), and 〈*τ*_*D*(*A*)_〉_*f*_ (Fig. 3c, lifetime image) is clearly reduced compared to 〈*τ*_*D*(*0*)_〉_*f*_. The same observations were made also for TGR5 variants Y111A and Y111F (Fig. 3c and SI Fig. 1b).

**Figure 4:**
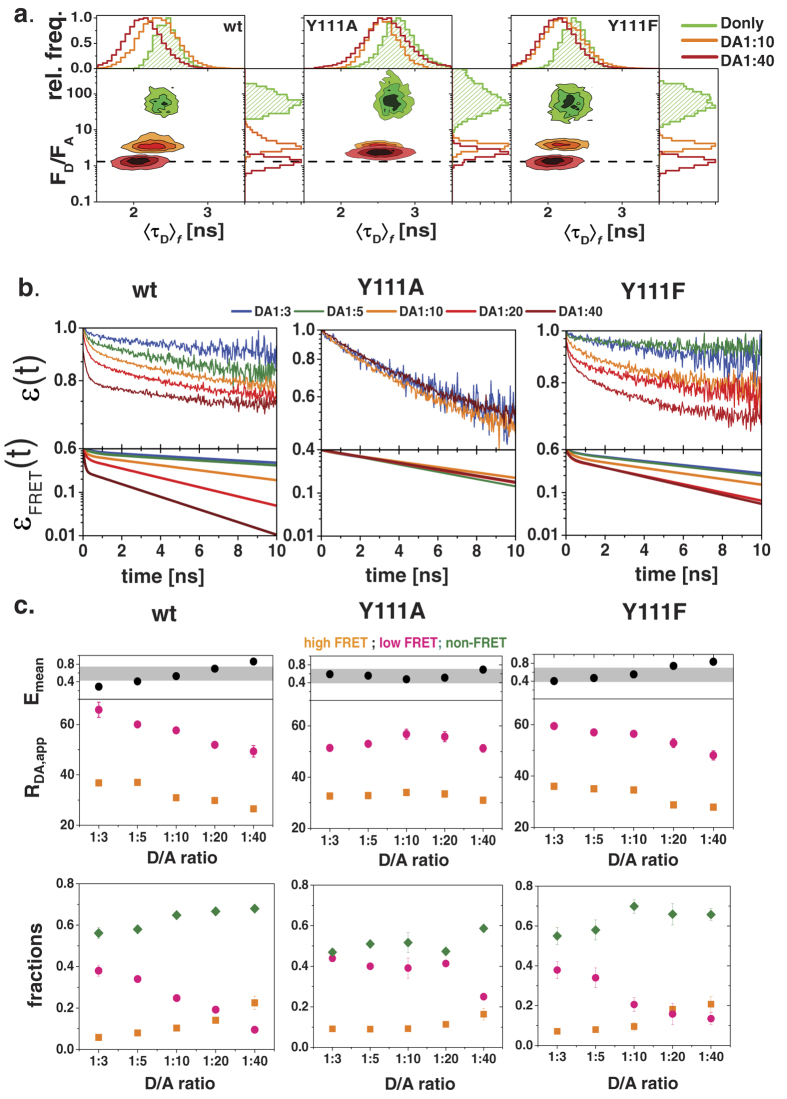
Pixel-integrated analyses of TGR5 FRET properties. (**a**) The MFIS-FRET 2D plots are generated with Origin 8.6 and show an overlay of two histograms of the (background, crosstalk and spectral shift) corrected fluorescence intensity ratio (*F*_*D*_*/F*_*A*_) vs. 〈*τ*_*D*(*A*)_〉_*f*_. TGR5 wt and TGR5 Y111F donors (in green) showed a 〈*τ*_*D*(*0*)_〉_*f*_ = 2.4 ns and a high green to yellow signal. With increasing amounts of the acceptor mCherry (orange and red islands) both parameters were strongly reduced in TGR5 wt and TGR Y111F, but not in TGR5 Y111A. All samples were corrected for relative brightness, relative direct mCherry excitation in the green detection channel, spectral shift of the Y111A variant, and background in the green and yellow channels (see methods 5.10 eqs 2 and 3). (**b**) FRET-induced donor quenching *ε*(*t*) derived from sub-ensemble fluorescence measurements on TGR5 variants at different donor-to-acceptor ratios. The time-axis measures the time between excitation and detection of donor photons. The upper row shows the experimental data. In the bottom row the offset (Non-FRET fraction) is subtracted and the result is termed *ε*_*FRET*_(*t*). In TGR5 wt and TGR5 Y111F, FRET clearly increased in a mCherry-dependent manner, whereas in TGR5 Y111A all *ε*_*FRET*_(*t*) curves behaved similar. (**c**) FRET-decays from sub-ensemble analysis at different donor-to-acceptor (D/A) ratios were fitted with a two-*k*_*FRET*_ fit to obtain two apparent distances *R*_*DA,1*_ and *R*_*DA,2*_ (upper row) with their corresponding FRET fractions (lower row) and to calculate the mean efficiency *E*_*mean*_. *E*_*mean*_ increased in an acceptor-dependent manner in TGR5 wt and TGR5 Y111F, whereas *E*_*mean*_ changed only slightly in TGR5 Y111A. These changes in *E*_*mean*_ correlate with a reduction of both apparent distances *R*_*DA,1*_ and *R*_*DA,2*_ in TGR5 wt and TGR5 Y111F: In the lower row, the *R*_*DA,1*_ fractions increase, whereas the *R*_*DA,2*_ fractions decrease in an acceptor-dependent manner. Orange: *R*_*DA,1*_ and *R*_*DA,1*_ fraction, pink: *R*_*DA,2*_ and *R*_*DA,2*_ fraction, green: non-FRET fraction, the grey bar in *E*_*mean*_ represents average *E*_*mean*_ for TGR5 Y111A.

The correlated FRET-specific change of both FRET-indicators is best visualized in a 2D-histogram plotting the ratio of the corrected fluorescence intensities of donor and acceptor (*F*_*D*_*/F*_*A*_) (SI Table S1) versus donor fluorescence lifetime (〈τ_*D*_〉_*f*_), where the color scale corresponds to the pixel frequency with black being highest (Fig. 4a). The correlated shift of both indicators proves the molecular proximity of TGR5 wt and TGR5 Y111A/F monomers suggesting the presence of at least homo-dimers. To study whether also higher order oligomers form, we performed acceptor titration experiments with varying donor to acceptor transfection levels resulting in an anticipated 40-fold higher acceptor concentration in the last titration step. Here, the FRET-indicators (*F*_*D*_*/F*_*A*_) and 〈*τ*_*D*(*A*)_〉_*f*_ allow for a qualitative interpretation of the measurements without applying a specific model. FRET senses the local proximity of binding partners within ~80 Å. Hence, if small oligomers exist 〈*τ*_*D*(*A*)_〉_*f*_ the fluorescence intensity ratio (*F*_*D*_*/F*_*A*_) will decrease with increasing acceptor concentration, whereas they do not change if only dimers exist. For TGR5 wt and TGR5 Y111F, 〈*τ*_*D*(*A*)_〉_*f*_ reduced significantly by 17% and 14%, respectively, whereas for TGR5 Y111A 〈*τ*_*D*(*A*)_〉_*f*_ reduced only by 7%. This behavior is also found in the fluorescence intensity ratios *F*_*D*_*/F*_*A*_. Here, significant transfection-level dependent FRET-changes are found for TGR5 wt and TGR5 Y111F, while only minor changes are found for TGR5 Y111A (Fig. 4a). The correlated shift of both FRET-indicators confirms that changes in FRET are indeed due to different concentrations. This suggests a significant formation of TGR5 wt and TGR5 Y111F oligomers but no or only few oligomers for TGR5 Y111A. We observed the distinct properties of TGR5 Y111A also via the fluorescence properties of the fused GFP, which was measured always as donor-only reference sample in the FRET experiments. While GFP fused to TGR5 wt and TGR5 Y111F had a fluorescence lifetime 〈*τ*_*D*(*0*)_〉_*f*_ = 2.4 ns, 〈*τ*_*D*(*0*)_〉_*f*_ increased to 2.8 ns in the Y111A variant (SI Fig. 1b). In addition to the lifetime shift, we found a spectral red shift of 13 nm in the emission spectrum of TGR5 Y111A excited at 488 nm as compared to TGR5 wt (SI Fig. 1c).

### TGR5 wt and TGR5 Y111F form higher-order oligomers, whereas TGR5 Y111A forms primarily dimers

The pixel-wise analysis of the fluorescence data by the fluorescence-averaged lifetime 〈*τ*_*D*_〉_*f*_ and the fluorescence intensity ratios (*F*_*D*_*/F*_*A*_) does not allow us to resolve multiple species because the information contained in the recorded fluorescence decays is reduced to two numbers. Hence, sample heterogeneities that naturally arise in imaging cannot be resolved. To overcome this limitation, the fluorescence decays are analyzed directly by pixel-integrated analysis with high precision. Here, two fluorescence decay curves *f*(*t*) are compared: the decay of a FRET sample *f*_*D*(*A*)_(*t*) and that of the donor-only reference *f*_*D*(*0*)_(*t*) (Fig. 4b). This comparison is conveniently done by computing the time-resolved FRET-induced donor decay *ε*(*t*), which is defined by the ratio of the two decays *f*_*D*(*A*)_(*t*)/*f*_*D*(*0*)_(*t*) as described in eq. (1). The supporting Figure 2 shows how *ε*(*t*) plots can be interpreted. The FRET-induced donor decay *ε*(*t*) allows visually identifying the population of all donor species. For instance, species with no-FRET give rise to a constant offset, while FRET-species cause decay. The slope of this decay in a semi-logarithmic plot as shown in Fig. 4b provides a measure of the rate constant of FRET, which increases with decreasing donor acceptor distance. A non-exponential decay indicates a mixture of distinct FRET species in which the donor and the acceptor are separated by different distances. The donor is quenched by all acceptors in its vicinity.

In Fig. 4b, the experimental fluorescence decays of all variants are displayed as *ε*(*t*) curves. Differences in the constant offset and the slope of the decays are clearly visible. For a better comparison of the slopes only the fraction of the FRET species was determined in a fit (equation (1), results see SI Table S2 and SI Figure 2) and displayed in Fig. 4b (*ε*_*FRET*_(*t*) curves). At a low donor to acceptor transfection level (DA 1:40), the decay has two distinct regions: a steep slope and a shallow slope region. The steep slope corresponds to a high rate constant of FRET, while the shallow slope corresponds to a low rate constant of FRET. For TGR5 wt and TGR5 Y111F, the slope depends on the transfection ratio, while no such dependency is observed for TGR5 Y111A.

To quantify these changes we formally describe the fluorescence decays by two FRET-rate constants, which are for convenience given in units of apparent distances *R*_*DA,app*_ (equation (5) and SI Table S2, SI Fig. 3). For all TGR5 variants, this *k*_*FRET*_ fit resulted in a short apparent distance *R*_*DA,app-1*_ with a small fraction and a long apparent distance *R*_*DA,app-2*_ with a large fraction. As shown in Fig. 4c, in TGR5 wt and TGR5 Y111F both apparent distances *R*_*DA,app-1*_ and *R*_*DA,app-2*_ became shorter (*R*_*DA,app-1*_ = 40–20 Å; *R*_*DA,app-2*_ = 75–50 Å) with increasing acceptor concentration. Furthermore, the species fractions also changed: the short distance-fraction increased from 7% to 30% in an acceptor-dependent manner, leading at the same time to a strong reduction of the long distance-fraction from 39% to 12%. We quantified this change by computing the mean energy transfer efficiency *E*_*mean*_ (equation (7)) of the FRET active species, which markedly increased for TGR5 wt and TGR5 Y111F in contrast to TGR5 Y111A. Considering TGR5 wt and TGR5 Y111F, the FRET efficiency changes significantly with the acceptor concentration (Fig. 4c), while this is not the case for TGR5 Y111A. Hence, the concentration of oligomers is very low for TGR5 Y111A, so that these data are best suited to study the structural features of the dimer.

Of note, to rule out any overexpression artifacts, we additionally considered proximity FRET using the titration experiments. Due to the single-molecule sensitivity of our confocal microscope, we could perform FRET experiments with acceptor concentrations of ~1 μM, which corresponds to a molecule density of < ~0.002 acceptor molecules/nm^2^ (see SI Notes). At these concentrations proximity FRET is negligible (E < 0.1)^34^.

### The TGR5 ligand TC has no influence on the oligomerization state of TGR5

**Figure 5:**
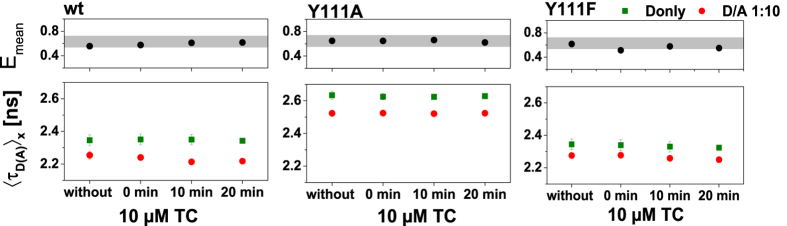
Influence on FRET after treatment with TGR5 ligand TC. HEK293 cells were transiently transfected with TGR5-GFP alone (Donly, green) or with TGR5-GFP and TGR5-mCherry at a ratio D/A 1:10 (DA, red). For time-series analysis three cells were selected using the Olympus time laps function, and MFIS-FRET measurements were taken before addition of 10 μM TC (without), immediately after addition of TC (*t* = 0), and after 10 min and 20 min, respectively. The species-averaged donor fluorescence lifetime 〈*τ*_*D*_〉_*x*_ was determined and plotted against time, as well as the mean efficiency *E*_*mean*_, which was calculated from data shown in SI Fig. 4. Each point represents the average of nine cells. No lifetime changes were observed for Donly samples and DA samples in the presence of the agonist TC.

It has been shown that activation by ligands can influence GPCR oligomerization^10^. To determine the ligand effect on TGR5, we tested whether taurocholate (TC) stimulation, a bile acid less cytotoxic than TLC in live cells, affects oligomerization of TGR5 wt, TGR5 Y111A, and TGR5 Y111F. A time series analysis was designed, where MFIS-FRET was measured in three cells before, directly after as well as 10 and 20 min after addition of 10 μM water soluble TC. We monitored FRET via the species-averaged donor fluorescence lifetime 〈*τ*_*D*(*A*)_〉_*x*_. As shown in Fig. 5, 〈*τ*_*D*(*A*)_〉_*x*_ was neither changed in donor samples (TGR5 GFP) nor in FRET samples (TGR5 GFP/mCherry). A more detailed FRET analysis of the time series experiments showed that neither the distances nor the species fractions changed markedly due to addition of TC (SI Fig. 4). These results indicate that TC does not influence the oligomerization state of TGR5 variants.

### Structural arrangement of homo-di- and oligomeric TGR5

Next, we analysed which structural features of the TGR5 complexes can be extracted from the observed FRET parameters. Previous studies by Sindbert *et al*.^35^ and Kalinin *et al*.^36^ have shown that the extent of FRET between two flexibly linked fluorescent probes can be accurately predicted by calculating the distance distribution between all fluorophore positions that are sterically accessible (accessible volume, AV) for a given structural model. As both fused fluorescent proteins have flexible connecting amino acid residues (SI Table 3) creating a large, widely distributed structural ensemble^37^, computer simulations generating probe distributions can be readily applied to study TGR5 assemblies by FRET.

### Simulation of the expected FRET properties

**Figure 6:**
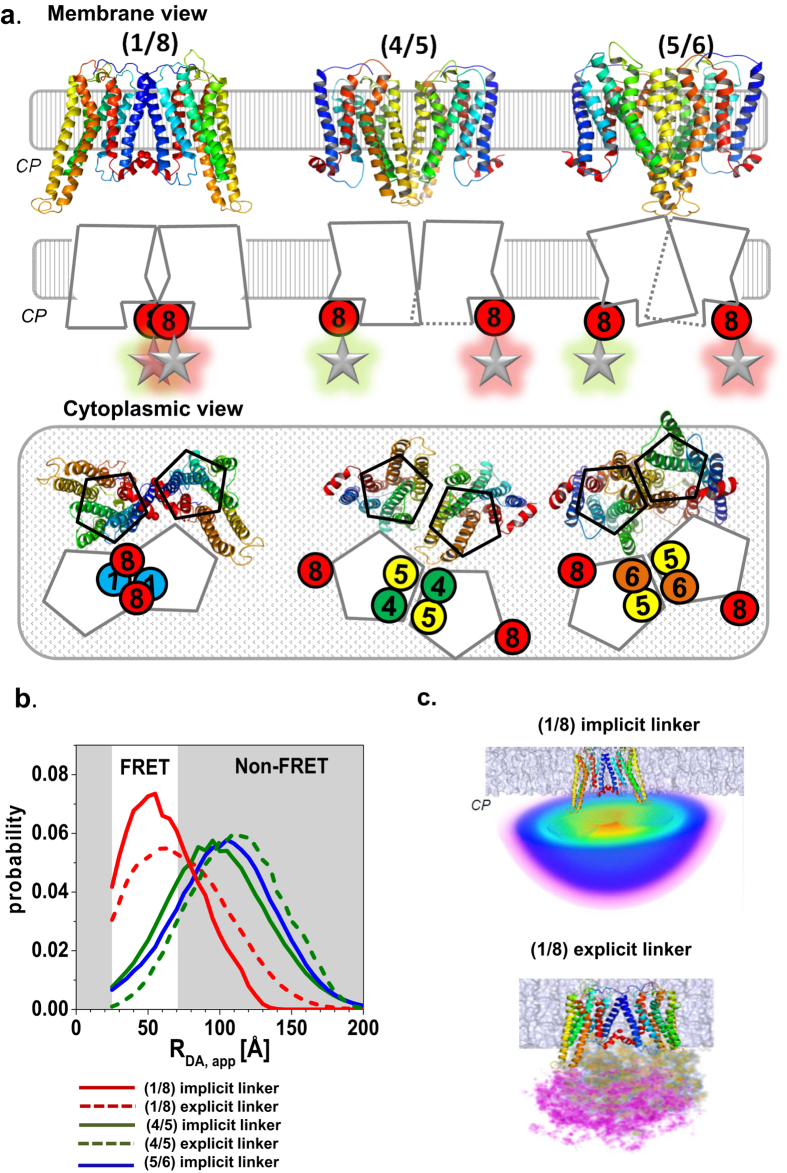
Homo-dimerization models and their distance distributions. (**a**) Homo-dimerization models with the following interfaces from left to right: (1/8), (4/5) and (5/6). TGR5 monomer helices are rainbow-coloured starting with TM1 in blue to H8 in red. Top row: membrane view of the interface models in cartoon and schematic representation (circles representing TMs). Bottom row: cytoplasmic view of the interface models. The fluorescent proteins, which are attached to the cytoplasmic H8, are presented as stars glowing in green for donor (eGFP) and red for acceptor (mCherry). Abbreviation: CP = cytoplasm. (**b**) Distance probability distributions calculated with an explicit (dotted line) and implicit linker (solid line) for the homo-dimerization models (1/8) (red), (4/5) (green), and (5/6) (blue). The non-FRET area is shaded in grey. (**c**) Positional distributions of the fluorescent probes for the TGR5 (1/8) interface. The implicit linker simulations yield weighted AVs for both fluorophores which overlap and create one huge sphere (top panel). The probability of the allowed fluorophore positions decrease from red, yellow over green, blue to pink. The explicit linker simulations yield a thermodynamic ensemble (bottom panel) depicted as an orange-blue and purple volume map, respectively. The ensembles also overlap to a high degree. Higher saturation represents higher fluorophore position occupancy. Both methods gave very similar results.

The structural model of the TGR5 monomer required for FRET modelling was generated by performing multi-template homology modelling based on seven template structures of related class A GPCRs (see SI methods “structural models of TGR5 dimers and tetramers” and ref. 38). As shown in Fig. 6a, we generated three possible homo-dimerization models with interfaces involving TM1-TM2-H8 (for convenience abbreviated as 1/8 dimer), TM4-TM5 (4/5 dimer), or TM5-TM6 (5/6 dimer). To assure accuracy, we compared two procedures for calculating the distance distributions between fluorophore positions for the TGR5 models: (i) Explicit linker simulations based on explicit peptide linker/GFP-MD-simulations followed by calculations of conformational free energies to weight each linker-GFP configuration in the presence of a TGR5 dimer and an implicit membrane bilayer (SI Fig. 5, see also SI methods). This thermodynamic ensemble (TE)-approach is expected to be more accurate than the following procedure but the computations are time consuming. (ii) Implicit linker simulations by AV-calculations weighted by a Gaussian chain distribution, so that entropic effects and geometric factors in terms of steric exclusion effects by the TGR5 oligomer and the membrane are taken into account (SI methods). The AV approach has to be calibrated to be accurate but it has the advantage that the computation is very fast.

The TE-approach results in a hemispherical arrangement of GFP on the cytoplasmic side, which is centred on the attachment point at helix 8 of TGR5 (SI Fig. 5) and each linker/GFP configuration is Boltzmann weighted according to the conformational free energy (SI Fig. 5). Configurations of lower probability are found when GFP approaches TGR5 due to energetically unfavourable contacts. The Boltzmann-weighted distribution of distances between the linker N-terminus and the GFP fluorophore shows a peak distance of about 45 Å, while the minimal distance is about 35 Å. This is due to the fact that the fluorophore is located 20 Å away from the linker C-terminus inside the β-barrel structure of GFP and thus is inaccessible to the linker’s N-terminus. The peak linker length (without considering GFP) is about 25 Å. This is about 5 Å longer than the average radius of gyration of a Gaussian chain polypeptide of the same number of residues (33 amino acids yielding 3.5 Å * 33^0.5^ = 20 Å^39^). The deviation shows that the linker with GFP does not exactly behave like a ‘perfect’ Gaussian chain. The Boltzmann-weighted fluorophore position map (Fig. 6c, SI Fig. 5) was used for inter-dye distance distribution calculations.

The implicit model (Fig. 6c) was tested as an alternative to account for dye-linker diffusion. The accessible volume (AV) approach was used to estimate all possible dye positions within the linker length from the attachment point without steric clashes with the macromolecular surfaces. The fluorophores are approximated by a sphere with a defined radius, which is estimated from the physical dimensions of the molecules (left panel). The connecting linker is modelled as a flexible cylinder. To take entropic effects into account, the linker was assumed to obey Gaussian chain behaviour. Thus, the fluorophore distribution density gradually drops as the distance from the attachment point increases. For the implicit model, the 55 amino acid residues (SI methods and SI Table S3) between the structured parts of the TGR5 C-terminus and GFP were considered as a flexible sequence with unknown structure with a length of ~203.5 Å at maximal extension.

Both methods for linker simulations gave very similar results. The (1/8) dimerization model shows a distance distribution between fluorophore positions between 25–150 Å with the highest probability at 55 Å and 60 Å for the explicit and implicit linker models, respectively. The distances between fluorophores in models (4/5) and (5/6) are similarly distributed with the highest probability at around 95–110 Å; i.e. the distance of most conformers is too large for significant FRET. Implicit and explicit linker models thus show very similar inter-dye distance distributions for all dimer models: The implicit model shows a 5 Å shift towards the higher length for the (1/8) dimer and a 15 Å shift towards the shorter length for the (4/5) dimer model.

Finally we can conclude that both linker simulation techniques predicted FRET and should distinguish a 1/8 dimer from 4/5 dimer and 5/6 dimer, respectively, because the FRET probe distance distributions have a characteristic peak at short distances (Fig. 6b). However, the FRET probe distance distributions of the two dimers involving TM5 are expected to be not distinguishable in our FRET experiments (Fig. 6b).

### In the first step of oligomerization contact sites in TM1 and helix 8 are involved

The shape of the distance distribution (determined by our linker simulation) and the concentration-dependent change in *E*_*mean*_ (using MFIS-FRET titration experiments) should allow us to distinguish (i) oligomerization interfaces and (ii) oligomerization pattern.

**Figure 7:**
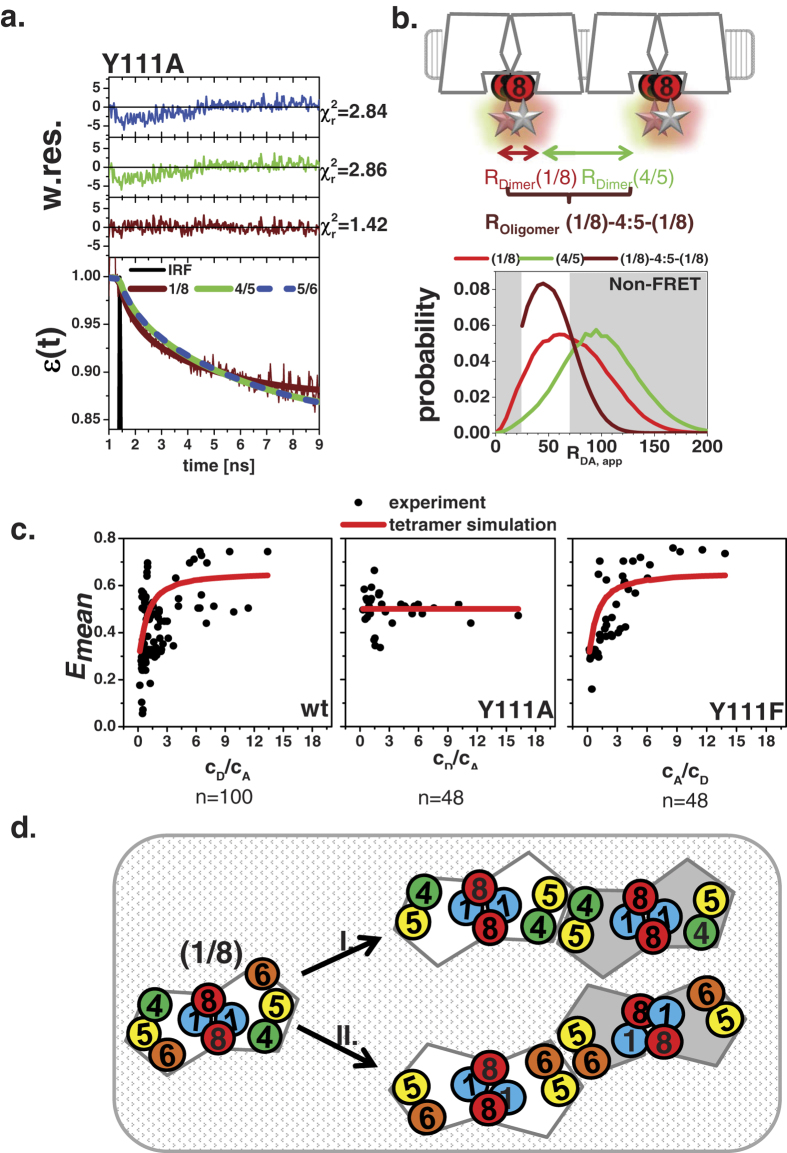
TGR5 oligomerization models. (**a**) Fit of the FRET-induced donor quenching curve *ε*(*t*) on TGR5 Y111A with two species normalized to unity: (i) Dimer (fraction x_Dimer_) with the complete distance distribution (FRET and Non-FRET) of the corresponding dimer models (Fig. 6b) and (ii) donor only/ FRET inactive molecules. Only the distance distribution of the 1/8 dimer model gives a satisfactory fit as judged by the weighted residuals and the reduced chi squared χ_r_^2^. Fit results of TGR5 Y111A for x_Dimer_: 1/8 dimer: 0.27; 4/5 dimer: 0.59; 5/6 dimer: 0.73. (**b**) The schematic presentation shows the two individual apparent distances from the interfaces (1/8) and (4/5). Both *R*_*Dimer*_ can be converted into FRET rates. In an oligomer the two FRET rates add up and have to be convolved to calculate the new apparent distance *R*_(*oligomer*)_. The resulting distance distribution is similar to the dimer (1/8). (**c**) Dependence of the TGR5 oligomerization monitored by the FRET efficiency (experiment (black) and modeled (red)) on the donor acceptor ratio c_A_/c_D_. In the cells the donor, acceptor and total TGR5 concentration (including inactive mCherry (30%))) varied between 0.25–6.3 μM, 0.1–5.0 μM and 0.5–13 μM, respectively.The dimer is composed of a donor acceptor distance of 45 Å, and the tetramer is composed out of two dimers separated by 100 Å. The modeled dissociation constant of the dimer K_D1_ was fixed to 10 nM for all TGR5 variants. The values for the modeled dissociation constants of the oligomer (Tetramer) were: K_D2_(TGR5 wt) = 70 nM, K_D2_(TGR5 Y111F) = 200 nM, K_D2_(TGR5 Y111A) = 2000 nM). (**d**) Two possible oligomers are reasonable I. ((1/8)-4:5-(1/8) and II. (1/8)-5:6-(1/8): TGR5 monomers form a dimer with the contact sites in TM1 (blue circle) and H8 (red circle). H8 is attached to fluorescent fusion proteins (GFP and mCherry). In a tetramer contact sites in TM4 (green circle) and TM5 (yellow) (**I**) or TM5 (yellow) and TM6 (orange) (**II**) create a second interface promoting a linear oligomer organization.

The concentration-independent FRET efficiency (Fig. 4) of the TGR5 Y111A variant suggests the preferential presence of homo-dimers. Therefore, it is a perfect variant to test which of our distance probe distributions describes the FRET-induced donor quenching curve *ε*(*t*) best. Figure 7a shows the fits using a model with the complete distance distribution (FRET and Non-FRET) of the corresponding dimer models (Fig. 6b, SI Table 4). Only the distance distribution of the 1/8 dimer model gives a statistically satisfactory fit as judged by the weighted residuals (*w. res*.) and the smallest *χ*_*r*_^2^. Hence, TM1 and helix 8 most likely form the primary oligomerization interface.

From the same titration experiments, we conclude that TGR5 wt and TGR5 Y111F are able to form higher-order oligomers because of the concentration-dependent increase in FRET efficiency (Fig. 4c). This finding implies that at least a second interface should exist for TGR5 homo-oligomer formation. As shown in Fig. 6b, the average apparent distances between fluorescent proteins attached to TGR5 helix 8 (without a coupled G-protein) were 120 Å for the (4/5) dimer model and 103 Å for (5/6) model, respectively, and the effective apparent oligomer distance for both patterns is approximately 49 Å (brown curve in Fig. 7b) due to the presence of multiple acceptors. We applied a dimer/tetramer simulation to our MFIS data to estimate the two corresponding association constants (Fig. 7c, SI Fig. 6) by analysing the dependence of the mean FRET efficiency *E*_*mean*_ on the ratio of donor to acceptor concentration (*c*_*D*_*/c*_*A*_). Moreover, the spread in the FRET efficiencies observed in Fig. 7c is also caused by the distinct protein concentrations in the cell and is taken into account in the simulations (SI Fig. 6a–c). For TGR5 wt and Y111F (K_D_ in 100 nM range), the simulations indicate that almost all dimers form tetramers, whereas TGR5 Y111A forms predominantly dimers (K_D_ in μM range).

## Discussion

We pursued a combined strategy applying cellular biology, MFIS-FRET, molecular modelling and simulations with a focus on dimerization and higher-order oligomerization of TGR5. We studied the influence of a mutation in the TGR5 ERY motif (TGR5 Y111A and Y111F) located in the transmembrane helix 3 (TM3) on oligomerization.

For our oligomerization studies we replaced the tyrosine residue in the highly conserved “D/ERY” motif in TM 3 and belongs to one of two clusters important for structural stability in GPCRs^40^. Mutation studies in Rhodopsin showed that the tyrosine (Y) mutation alone did not or only marginally affect receptor function^41^ regarding receptor expression, G-protein binding and ligand affinity in contrast to the residues D/ER. Consistent with literature results^41^, the TGR5 Y111 variants, Y111A and Y111F, were normally localized at the plasma membrane and activated by both bile acid agonists TLC and TC to a level comparable to TGR5 wt. These findings implicated no obvious impaired ligand binding affinities or G-protein coupling. However, we observed significant differences in oligomer formation between Y111A and Y111F as assessed by Co-IP experiments and FRET measurements in live cells.

As the overall protein concentrations are very low (1–7 μM), we can rule out any overexpression artifacts due to proximity FRET (see SI notes). Therefore our MFIS-FRET titration data are best described with models assuming formation of the 1/8 dimer as the first step in oligomerization (Fig. 7a). In the second step, we suggest that TM5 (Fig. 7d) is involved as known from other oligomerization models of class A GPCRs^15^,^19^,^20^. According structural models were as templates for predicting the distance distributions in Fig. 7b. As shown in Fig. 7d and SI Fig. 7, oligomer array configurations^15^,^19^,^20^ either could have a row or a rhomboid tetramer organization. One might assume that TGR5 oligomers most likely resemble in a one-dimensional row-like array mediated by a single oligomerization interface, because a single mutation in the ERY motif, Y111A in TM3, affects the association significantly (factor 10).

**Figure 8:**
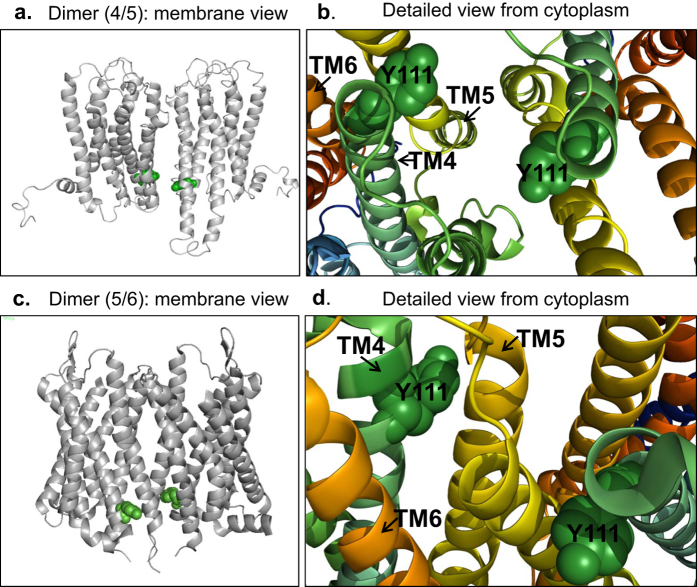
Influence of the Y111 residue on oligomerization. (**a,c**) The dimerization models of the (4/5) and (5/6) interface are displayed as a grey colored cartoon viewed from the membrane. Residue Y111 located in TM3 is depicted as a green sphere in each TGR5 monomer. (**b,d**) Blow-up of the region around residue Y111 to show possible interactions between Y111 from one TGR5 molecule with residues in TM4 (green), TM5 (yellow) and TM6 (orange) in a second TGR5 molecule.

As shown in Fig. 8, the Y111 residue can interact with TM4-TM5 or TM5-TM6 dependent on the oligomerization. In both cases, the potential interaction sites involving TM5 can be affected during oligomerization. This observation is supported by two crystal structures: In the (4/5) model, as shown in CXCR4 (PDB ID: 3ODU), a charge-assisted interaction between Y111 and R146 (TM4) is possible; likewise an interaction is possible in the (5/6) model between Y111 and R280 (helix 8), as shown in the μ-opioid receptor (PDB ID: 4DK2).

It was reported that GPCR oligomerization could be affected by ligand binding^10^, therefore we addressed this question in a time-series FRET analysis by ligand stimulation with TC. From simulation experiments, we expect that after G-protein binding the average apparent distances between TGR5-GFP and TGR5-mCherry get longer. Effective oligomers distributions with and without G-protein are indistinguishable, because a distance distribution difference of less than 8 Å is smaller than the anticipated accuracy of the models (see SI Table 5). In fact, the MFIS-FRET measurements showed no change in FRET properties after TC treatment, an observation that is also supported by literature^22^. As an indicator of G-protein binding, we successfully proved cAMP increase after ligand treatment in all TGR5 variants, which has also been shown recently^9^,^42^,^43^. We have no evidence that TGR5 oligomerization is affected by ligand treatment and subsequent G-protein coupling.

It is not too surprising that G protein activation does not change when reducing TGR5 higher oligomer formation, because rhodopsin and β-AR receptors in a monomeric, dimeric and oligomeric state, respectively, are capable to activate the respective G-protein^11^,^14^,^44^,^45^. Moreover, as described by Scarselli *et al*.^46^, PALM experiments using a class A GPCR suggested that oligomerization remains unchanged by the addition of the agonist. This is in line with our findings for our class A receptor TGR5 and the bile acid ligands. While the function of higher-order oligomers for most GPCRs is still unknown, identification of dimer/oligomer interfaces will allow for targeted disruption of dimer/oligomer formation and thus elucidation of the biological relevance of these complexes. This has just been demonstrated for rhodopsin where disruption of dimerization with small peptides decreased receptor stability^44^. We recently showed that the loss of α-helicality in the TGR5 C-terminus, which constitutes the major interaction surface in the 1/8 interface, severely impairs TGR5 membrane localization and activity^9^. One can thus speculate that this influence on membrane localization and activity results from a distorted TGR5 dimerization in the ER. Additionally, the design of bivalent ligands targeting a homodimer can reduce off-target effects caused by the transactivation or inhibition of GPCRs in heterodimers^47^. Knowledge of the primary dimerization interface of a GPCR can guide the development of such bivalent ligands. The discovery that TGR5 forms higher order oligomers and that Y111 is important for this process thus is the first step for deciphering and modulating the functional relevance of TGR5 oligomerization.

To conclude, TGR5 wt forms homo-oligomers. Dimerization involves interaction contact sites in TM1 and helix 8, while its oligomerisation additionally involves TM5. Both modelled patterns, (1/8)-5:6-(1/8) and (1/8)-4:5-(1/8), are currently possible with Y111 forming charge-assisted and/or polar interactions with residues within the mentioned interfaces.

## Methods

### Multiparameter Fluorescence imaging spectroscopy (MFIS)

All measurements in live cells were performed on an inverted confocal laser scanning microscope (FV1000 Olympus, Hamburg, Germany) additionally equipped with a single photon counting device with picosecond time-resolution (Hydra Harp 400, PicoQuant, Berlin, Germany) with home built extensions for MFD as described in^28^. Using a 60x water immersion objective (Olympus UPlanSApo NA 1.2) the sample was excited with selected wavelengths (GFP at 488 nm with 400 nW, mCherry at 559 nm with 650 nW) of a NCH white light laser with a pulse-repetition rate of 40 MHz. The emitted light was collected and separated into its parallel and perpendicular polarization and into its green and red component (beam splitter 595DCLX, AHF, Germany). GFP fluorescence was then detected by single photon avalanche detectors (PDM50-CTC, Micro Photon Devices, Bolzano, Italy) in a narrow range of its emission spectrum (bandpass filter: BS 520/35, AHF, Tübingen, Germany). mCherry fluorescence was detected by cooled hybrid detectors (HPMC-100–40, Becker&Hickl, Berlin, Germany, with custom designed cooling), of which the detection wavelength range was set by the bandpass filters HC 607/70 (AHF). MFIS images were generated via raster-scanning the sample in a continuously moving beam manner. Images were taken with 20 μs pixel dwell time and a resolution of 103 nm per pixel. With 488 nm excitation, series of 40 frames were merged into one image; with 559 nm excitation, series of 20 frames were merged together. Images were further analyzed using custom-designed software available from our homepage (http://www.mpc.hhu.de/software.html). Description of sample preparation and microscope calibration can be found in the SI methods 1 and 2.

### Pixel-integrated, time-resolved ε(t) illustration

To identify appropriate pixel in the cells for further pixel-integrated analysis, we computed all fluorescence parameters for each pixel and selected the pixels in 2D-histograms of several FRET indicators (see SI methods 2 pixel-wise analysis). A pixel population with homogeneous properties was selected and then integrated for subsequent pixel-integrated sub-ensemble analysis. The time-dependent FRET parameter *ε*(*t*) contains information on the underlying FRET-rate distribution and is proportional to the probability that FRET occurs at a certain time. After pixel selection, *ε*(*t*) was plotted for direct visualization of molecular species with different FRET efficiencies in sub-ensemble data. *ε*(*t*) is calculated as the ratio of normalized fluorescence decays of the FRET sample *f*_*D*(*A*)_(*t*) and donor-only sample, *f*_*D*(*0*)_(*t*) (see eqs 3, 4).


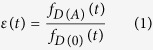



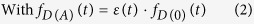


*ε*(*t*) is the probability density function of the occurring FRET governed by FRET rate constant(s), *k*_*FRET*_. The decaying part of *ε*(*t*) represents the features of FRET: high- or low-FRET can be directly read out from the decay slope. The amplitude of the decaying part indicates the FRET-active species fraction, *x*_*FRET*_. Accordingly, the offset of *ε*(*t*) is the FRET-inactive fraction, (1 − *x*_*FRET*_).

### Pixel-integrated MFIS-FRET analysis using *k*_*FRET*_ models

To determine FRET parameters from pixel-integrated, sub-ensemble data the reference samples were fitted by a multi-exponential relaxation model accounting for a multi-exponential fluorescence decay of the donor in the absence of FRET:


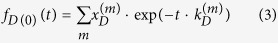


in which m = 3 considers that FPs in living cells usually show at least a bi-exponential characteristic^32^. Fit parameters in donor decay include three normalized pre-exponential factors 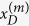 (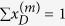) and three decay rate constants 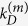, which are the reciprocals of fluorescence lifetimes. The quenched donor decay *f*_*D*(*A*)_(*t*) is given by:


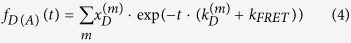


and *k*_*FRET*_ is the FRET rate constant. The fitted parameters in the 1 − *k*_*FRET*_ model are *x*_*FRET*_ and *k*_*FRET*_.

From the *ε*(*t*) diagrams it’s clear that our data have to be fitted with *m* = 2, then we say it’s a two-state model, from which we obtain two FRET rate constants and therefore two apparent distances. The quenched donor decay *f*_*D*(*A*)_(*t*) in eq. 4 is now extended:


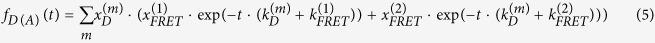



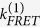
, 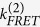 are the FRET rate constants and FRET species fractions, 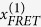, 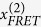. In the FRET-samples molecules not performing FRET are considered as No-FRET fraction. Each FRET rate constant is converted to an apparent distance 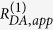



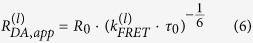


in which the unquenched GFP fluorescence lifetime is *τ*_*0*_ = 2.4 ns and the Förster radius between GFP and mCherry is *R*_*0*_ = 52 Å (including static κ^2^ = 0.476).

### Mean energy transfer efficiency

The mean (steady-state) transfer efficiency *E*_*mean*_ is obtained using the FRET fractions and the apparent distances (*R*_*DA,app*_) obtained from eq. 6.


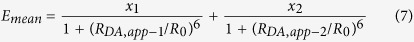


### Effective energies of linker/GFP conformations in the presence of TGR5 dimers and an implicit membrane

Molecular dynamics simulations of GFP bound to a linker have been performed as detailed in the SI methods. Snapshots of the MD simulations of the linker/GFP construct extracted in intervals of 50 ps were stripped of water molecules and ions, and the principle axis with the lowest moment of inertia of the first residue of the linker was aligned along the z-axis. The snapshots were then rotated in steps of 90° around the z-axis to increase the sampling density and subsequently placed in proximity to residue 295 of either TGR5 monomer for any of the TGR5 dimers (1/8 interface; 4/5 interface) (SI Fig. 4). For each snapshot, the effective conformational energy *E*_effective, conf_ (i.e., the sum of gas phase energy and solvation free energy) was computed using the FEW^mem^ program^48^,^49^, with the TGR5 dimers embedded in an implicit membrane of 34 Å width and using dielectric constants of 34, 4, and 1 for the outer to inner membrane slabs with a width of 5, 6, and 6 Å, respectively (SI Fig. 4)^50^,^51^; for water and protein, dielectric constants of 80 and 1 were used, respectively. The counter ion concentration for the APBS calculation^52^ was set to 0.15 mM. For all other parameters, default values as set in FEW^mem^ were used. All snapshots in which GFP penetrated the membrane, or in which GFP or the linker clashed with the TGR5 dimer, were omitted, leaving ~10.000 snapshots for the analysis. The distribution of the C-alpha atom of the central residue of the fluorophore from these snapshots shows that GFP essentially moves within a hemisphere on the cytosolic side of the membrane beneath the dimer (SI Fig. 4).

### Thermodynamic Ensemble (TE) using explicit linker/GFP configurations

From the explicit linker/GFP configurations, the thermodynamic ensemble (TE)-distribution is computed as a weighted average of the linker distance. The weights were determined according to a Boltzmann distribution


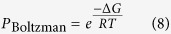


*R* is the gas constant, *T* is 300 K, and Δ*G* is the difference between the Gibbs energy of the current snapshot and the energetically most favorable one. *G* is determined as the difference between *E*_effective, conf._ (see section above) and the contribution from the configurational entropy *S*


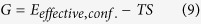


We assumed that *S* is dominated by the configurations of the linker, whereas configurations of GFP are assumed to provide no contribution. This seems justified given that GFP is structurally much more stable than the linker: the linker largely consists of the TGR5 C-terminus, a part of GPCRs that has either been not fully resolved in any GPCR structure due to its high flexibility^53^,^54^,^55^ or, when resolved in small parts, shows random coil formation^56^. Thus, we considered the linker a random hetero-polymer for which low energy conformations can structurally vary largely. Therefore, a random energy model^57^ was used to describe its energy landscape. According to the random energy model, the entropy of a configuration with a given *E*_effective, conf._ is^57^


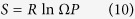


with Ω being the overall number of conformational states. The probability of occurrence *P* for each energy state is obtained from


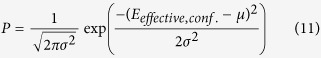


with *μ* being the mean and *σ* the standard deviation of the frequency distribution of *E*_effective, conf._. The assumption underlying eq. 11 is that the energy is Gaussian distributed^57^, which is approximately fulfilled in our case (data not shown).

MM-PBSA calculations show a range of *E*_effective, conf._ of several hundred *kcal mol*^*−1*^ for proteins of sizes similar to that used in the present study^58^,^59^. In agreement with this, *E*_effective, conf._ computed for the linker/GFP configurations attached to the TGR5 dimer spans a range of ~1.000 *kcal mol*^*−1*^. However, such an energy range would lead to unrealistically low probabilities for the higher energy configurations. We thus linearly scaled *E*_effective, conf._ such that the linker/GFP configuration with the highest energy has a probability of occurrence in a Boltzmann distribution of 1/Ω (SI Fig. 4). Finally with the scaled energies, *P* (eq. 11), *S* (eq. 10), and *G* (eq. 9) were calculated, and from these the weights according to eq. 8 for the weighted average of distances between 35 and 90 Å (SI Fig. 4).

To conclude, the TEs were constructed by explicit peptide linker/GFP MD simulations followed by calculations of conformational free energies (eqs 8, 9, 10, 11) to weight each linker-GFP configuration. In the TE approach, the weights of the points obtained from the explicit linker model were used to assign the weights of the inter-probe distances.

## Additional Information

**How to cite this article**: Greife, A. *et al*. Structural assemblies of the di- and oligomeric G-protein coupled receptor TGR5 in live cells: an MFIS-FRET and integrative modelling study. *Sci. Rep.* **6**, 36792; doi: 10.1038/srep36792 (2016).

**Publisher’s note:** Springer Nature remains neutral with regard to jurisdictional claims in published maps and institutional affiliations.

## Supplementary Material

Supplementary Information
