# Supplementary material for: Structural assemblies of the di- and oligomeric G-protein coupled receptor TGR5 in live cells: an MFIS-FRET and integrative modelling study
Source: Sci Rep. 2016 Nov 11;6:36792. doi: 10.1038/srep36792 (PMC5105069; doi:10.1038/srep36792)
Supplement: Supplementary Information [file srep36792-s1.pdf]

Supporting Information

Structural assemblies of the di- and oligomeric G-protein coupled receptor TGR5 in live cells: an MFIS-FRET and integrative modelling study

[1]Annemarie Greife, [1]Suren Felekyan, [1]Qijun Ma, [2]Christoph G.W. Gertzen, [3]Lina Spomer, [1]Mykola Dimura, [1]Thomas O. Peulen, [3]Christina Wöhler, [3]Dieter Häussinger, [2*]Holger Gohlke, [3*]Verena Keitel, [1*]Claus A.M. Seidel

[1] Chair for Molecular Physical Chemistry, Heinrich Heine University Düsseldorf, 40225 Düsseldorf, Germany;

[2] Institute for Pharmaceutical and Medicinal Chemistry, Heinrich Heine University Düsseldorf, 40225 Düsseldorf, Germany;

[3] Clinic for Gastroenterology, Hepatology and Infectious Diseases, Heinrich Heine University Düsseldorf, 40225 Düsseldorf, Germany

*Corresponding authors: C.A.M. Seidel, H. Gohlke, V. Keitel

Content

Supporting Tables p.3-7

Supporting Figures p.8-17

Supporting Notes p.18

Supporting Methods p.19-33

Glossary p.34-35

Supporting References p.36-37

Supporting Tables

Supporting Table 1: Parameters for determination of the corrected green to yellow fluorescence intensity ratio F_D/F_A necessary for the 2D histograms. The background B was determined from untransfected cells. The green to yellow fluorescence intensity ratio (F_D/F_A) was corrected for crosstalk (characterized by the crosstalk factor α), background $\langle B \rangle$, detection efficiencies of D (g_G) and A (g_Y). The acceptor fluorescence used for 2D-FRET must also be corrected for additional direct acceptor excitation *DE* and relative concentration dependent brightness DE_{rel}. All samples were corrected for distinct fluorescence quantum yields Φ and a spectral shift factor γ (especially for TGR5 Y111A) which is considered in the corrected green detection efficiency (g_G*).

	α	$\langle B_G \rangle$ [kHz]	$\langle B_Y \rangle$ [kHz]	γ	Φ	DE_{rel} [kHz]
**wt**	0.09	0.3	1	1	1	DA1:10= +0.76<br>DA1:40= +3.78
**Y111A**	0.28	0.3	2	0.61	1.125	DA1:10= +4.28<br>DA1:40= +0
**Y111F**	0.1	0.3	1	1	1	DA1:10= +1.76<br>DA1:40= +3.02

Supporting Table 2: Parameters for *ε(t)* diagram in **Fig. 4** for each TGR5 variant. The parameters b0-b4 are obtained from the fit equation $f = b_0 + b_1 \cdot e^{-\frac{x}{b_2}} + b_3 \cdot e^{-\frac{x}{b_4}}$. b_0 determines the Non-FRET fraction (Donly fraction), b_1 and b_3 are the two FRET fractions and b_2 and b_4 are the corresponding decay times. **Supporting Figure 2** shows how to generate and interprete *ε(t)* diagrams.

**TGR5**	**Fit**	**DA1:3**	**DA1:5**	**DA1:10**	**DA1:20**	**DA1:40**
**wt**	b_0:	0.90	0.84	0.89	0.81	0.82
	b_1:	0.01	0.04	0.03	0.04	0.04
	b_2:	0.30	0.18	0.23	0.33	0.46
	b_3:	0.09	0.13	0.08	0.16	0.15
	b_4:	5.70	8.04	1.89	3.80	5.12
**Y111A**	b_0:	0.42	0.42	0.45	0.43	0.43
	b_1:	0.02	0.01	0.19	0.03	0.03
	b_2:	0.73	0.63	5.28	0.56	0.72
	b_3:	0.55	0.56	0.36	0.55	0.55
	b_4:	6.09	5.02	5.26	4.48	4.90
**Y111F**	b_0:	0.73	0.74	0.69	0.68	0.77
	b_1:	0.11	0.06	0.18	0.09	0.02
	b_2:	5.73	2.7	3.88	2.30	0.43
	b_3:	0.15	0.19	0.13	0.23	0.21
	b_4:	771.45	90.75	765.84	24.83	2.80

Supporting Table 3: Sequence Information for AV-simulation. Untranslated region, TGR5 coding sequence, linker and GFP (4EUL) or mCherry (2H5Q) sequence of the analyzed TGR5 variants are summarized and used for TGR5 dimer and oligomer simulations. The position of the Y111 residue in the ERY motif for mutagenesis is highlighted. Sequences with unknown secondary or tertiary structures are underlined and are kept flexible in AV simulations.

	**TGR5 wt-FP**
5'UTR	none
TGR5	MTPNSTGEVPSPIPKGALGLSLALASLIITANLLLALGIAWDRRLRSPPAGCFFL<br>SLLLAGLLTGLALPTLPGLWNQSRRGYWSCLLVYLAPNFSFLSLLANLLLVH<br>GER**Y**MAVLRPLQPPGSIRLALLLTWAGPLLFASLPALGWNHWTPGANCSSQA<br>IFPAPYLYLEVYGLLLPAVGAAAFLSVRVLATAHRQLQDICRLERAVCRDEPS<br>ALARALTWRQARAQAGAMetLLFGLCWGPYVATLLLSVLAYEQRPPLGPGTL<br>LSLLSLGSASAAAVPVAMetGLGDQRYTAPWRAAAQRCLQGLWGRASRDSP<br>GPSIAYHPSSQSSVDLDLNY
Cloning Linker	GSTGRH
GFP (4EUL)<br>= donor (D)	MVSKGEELFTGVVPILVELDGDVNGHKFSVSGEGEGDATYGKLTLKFICTTG<br>KLPVPWPTLVTTFGYGLQCFARYPDHMKQHDFFKSAMPEGYVQERTIFFKDD<br>GNYKTRAEVKFEGDTLVNRIELKGIDFKEDGNILGHKLEYNYNSHNVYIMAD<br>KQKNGIKVNFKIRHNIEDGSVQLADHYQQNTPIGDGPVLLPDNHYLSYQSALS<br>KDPNEKRDHMVLLEFVTAAGITLGMDELYK*
mCherry<br>(2H5Q)<br>= acceptor (A)	MVSKGEEDNMAIIKEFMRFKVHMEGSVNGHEFEIEGEGEGRPYEGTQTAKLK<br>VTKGGPLPFAWDILSPQFMYGSKAYVKHPADIPDYLKLSFPEGFKWERVMNF<br>EDGGVVTVTQDSSLQDGEFIYKVKLRGTNFPSDGPVMQKKTMGWEASSERM<br>YPEDGALKGEIKQRLKLKDGGHYDAEVKTTYKAKKPVQLPGAYNVNIKLDIT<br>SHNEDYTIVEQYERAEGRHSTGGMDELY*

Supporting Table 4: Comparison of results from different fit models. Donor fluorescence lifetime decay histograms in presence of acceptor in TGR5 variants are fitted based on *2k-FRET* and AV simulated distance distributions for different dimer (1/8), (4/5) and oligomer (1/8)-4:5-(1/8) interfaces with only one fit parameter – Donly fraction. Interface dimer (4/5) yields very low Donly fractions compared to all other models: it is known that ~30 % of mCherry acceptor dyes are not active in cells; hence at least ~30 % Donly fractions are expected. Based on this we concluded that interface dimer (4/5) as the primary dimer interface in TGR5 variants are less likely. wt[1] is DA1:3, all other DA ratios are 1:20.

	**wt[1]**		**wt**		**Y111A**		**Y111F**	
	Donly fraction	X^2	Donly fraction	X^2	Donly fraction	X^2	Donly fraction	X^2
Two k_{FRET}	0.74	*1.61*	0.64	*1.59*	0.69	*1.39*	0.62	*1.61*
dimer (4/5)	0.43	*1.65*	0.00	*1.83*	0.00	*4.94*	0.14	*1.54*
dimer (1/8)	0.82	*1.91*	0.67	*2.68*	0.58	*2.61*	0.74	*1.83*
oligomer	n.d	*n.d*	0.74	*2.82*	0.65	*2.86*	0.78	*1.88*

Supporting Table 5: Overview of the mean distances ⟨R_{DA}⟩ calculated for the possible tetramer models with or without G-Protein. The primary interfaces for dimerization are in brackets (x/x) and secondary interfaces for oligomerization are abbreviated –x:x-. The numbers are the corresponding (transmembrane) helices involved in binding interactions. The apparent mean distances ⟨R_{DA}⟩$_{app}$ of each label pair involved in dimerization are bold. A schematic presentation of the tetramer models is shown in **Supporting Figure 4**. For example in the model (1/8)-4:5-(1/8) (with G-Protein always determined as label C, even when it is absent) label pairs A-E and B-D with the primary interfaces (1/8) show a distance 59-66 Å measured between the fluorescent proteins attached to helix 8, and the label pair A-B with the secondary interface -4:5- shows a mean distance of 133 Å. Further calculated distances in this oligomer are measured from label pairs A-D, B-E, B-D and are comparable to the distances obtained from A-B.

	**⟨R_{DA}⟩, [Å]**					
	**with G-Protein**			**without G-Protein**		
**Label pair**	**(1/8)-4:5-(1/8)**	**(1/8)-5:6-(1/8)**	**(5/6)-4:5-(5/6)**	**(1/8)-4:5-(1/8)**	**(1/8)-5:6-(1/8)**	**(5/6)-4:5-(5/6)**
**A-B**	133	119	**128**	98	94	**103**
**A-D**	134	**63**	128	97	**64**	120
**A-E**	**66**	129	65	**58**	93	72
**B-D**	**59**	108	64	**57**	91	69
**B-E**	131	**80**	116	99	**71**	106
**D-E**	136	123	**104**	101	94	**103**

Supporting Figures

Supporting Figure 1

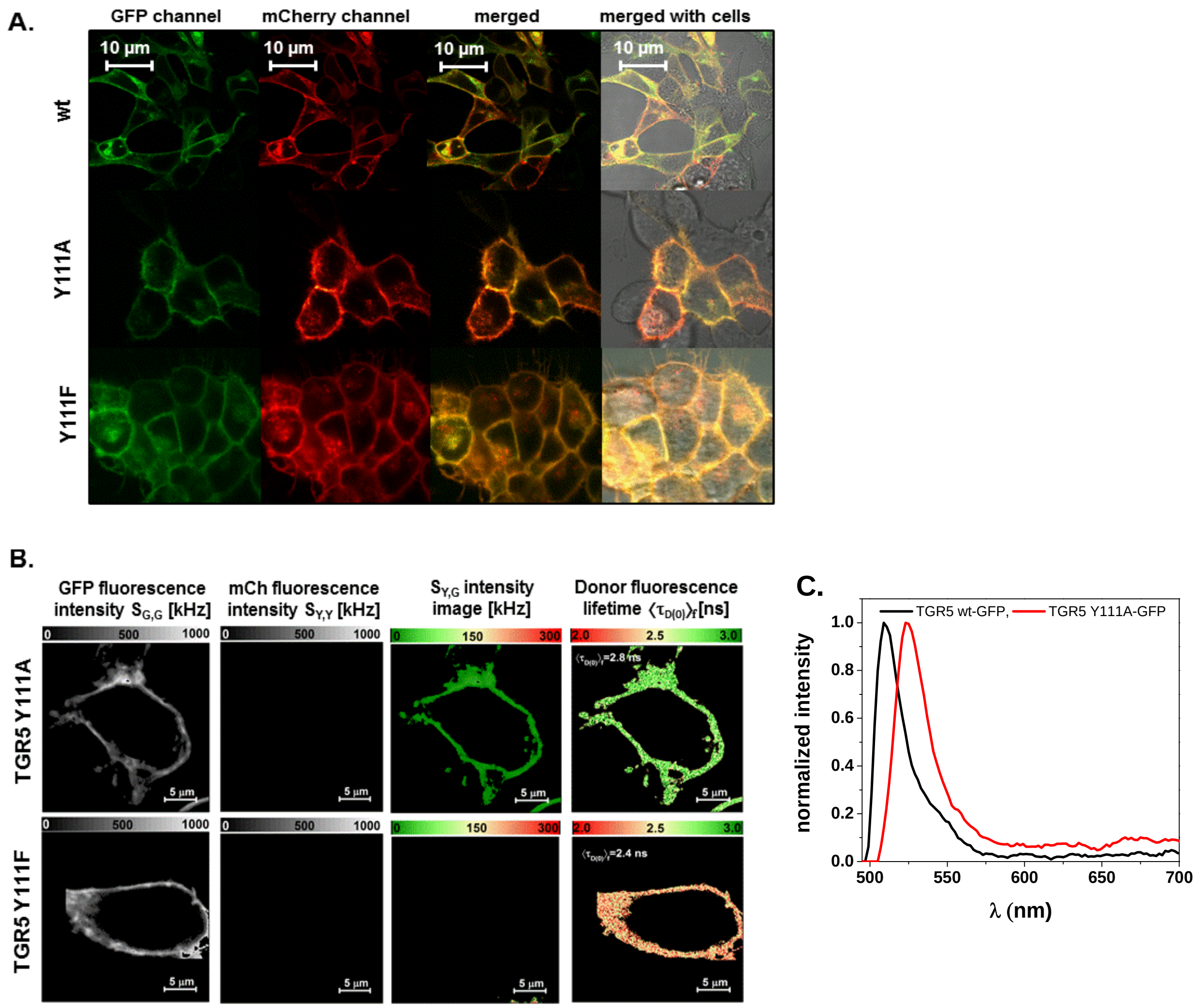

Supporting Figure 1: Live cell imaging and MFIS analysis of TGR5 donors

(a) HEK293 cells, transiently transfected with TGR5-GFP and TGR5-mCherry (transfection ratio 1:10), were imaged for co-localization of GFP and mCherry using sequential scanning and a scanning resolution of 1024 x 1024 pixels. Each TGR5-GFP and TGR5-mCherry picture is shown in a false color saturation mode and then overlaid by using green and yellow intensity colours. TGR5-GFP and TGR5-mCherry wt, Y111A and Y111F (from top to bottom) are clearly co-localized at the cell membrane. Scale bar 10 µm. **(b)** MFIS analysis of TGR5 transfected

HEK293 cells by comparing (from left to right and top to bottom row) the GFP fluorescence intensity, mCherry fluorescence intensity, the donor fluorescence lifetime $\langle\tau_{D(0)}\rangle_f$, and mCherry photons after excitation of GFP ($S_{Y,G}$). The fluorescence-averaged donor lifetime in the absence of an acceptor $\langle\tau_{D(0)}\rangle_f$ in the Y111A variant is 2.8 ns compared to 2.4 ns for Y111F. The presence of green photons in the yellow channel is due to a higher crosstalk, background and red shift in the Y111A variant. **(c)** GFP was excited at 488 nm and emission spectrum was measured from 495 nm to 700 nm in a 2 nm step size and a 2 nm spectral band width at Olympus FluoView1000 microscope. TGR5 wt-GFP shows the typical emission maximum at 510 nm, whereas TGR5 Y111A-GFP shows a 13 nm red shift towards 523 nm. Three cells for each curve were measured, the background was subtracted and the average intensity normalized to the maximum. The Y111A MFIS data were corrected for the spectral shift.

Supporting Figure 2

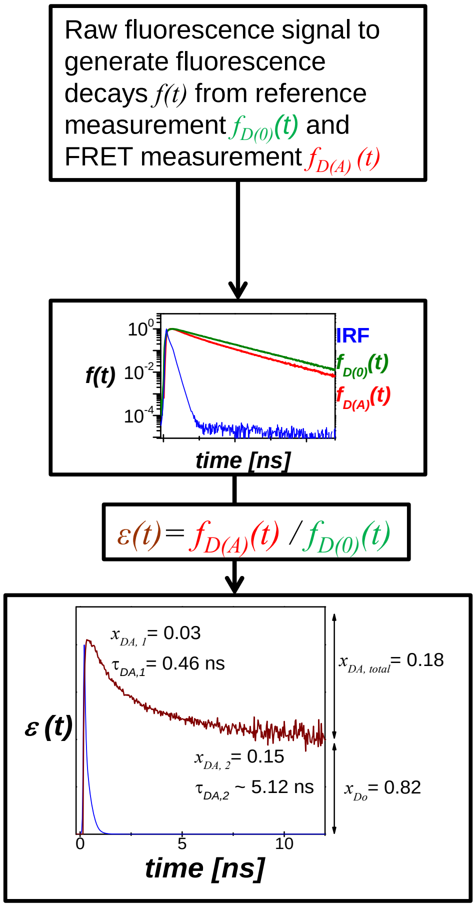

Supporting Figure 2. Guideline for presentation and interpretation of *ε(t)* diagrams: In the first step, the raw fluorescence signal decays *f(t)* from the reference measurement $f_{D(0)}(t)$ (green) and from the FRET measurement $f_{D(A)}(t)$ (red) are selected and corrected with the instrument response function curve (*IRF*, blue) for a time shift. In the second step, the $f_{D(A)}(t)$ decay is divide through $f_{D(0)}(t)$ decay (**eqation (1) in main text**). The resulting decay $\varepsilon(t)$ is normalized to 1 and plotted versus time in ns. As example the TGR5 wt DA1:40 experiment is used. In this case the non-FRET fraction x_D (b0 in Table S2) is 0.82. The corresponding total FRET fraction $x_{DA,total}$ is 0.18. As the decay is clearly bi-exponential, two FRET fractions $x_{DA,1}$, $x_{DA,2}$ (b1, b3) and the corresponding fluorescence lifetimes $\tau_{DA,1}$, $\tau_{DA,2}$ (b2, b4) are resolvable by the equation used in Table S2.

Supporting Figure 3

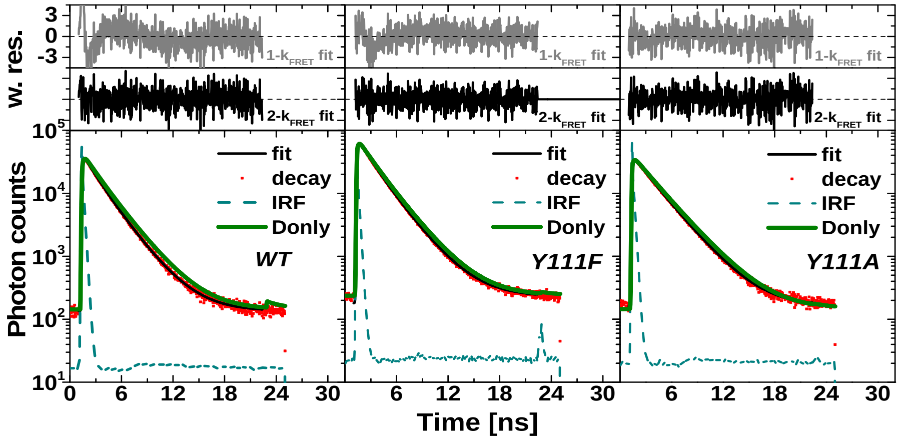

Supporting Figure 3: Fit fluorescence decays with different models for TGR5 variants.

Fitting the sub-ensemble fluorescence decays of the FRET samples (DA1:10) with k_{FRET} models showed that two FRET rates are necessary to fit these data accurately. The decays of Donly (TGR5-GFP) and FRET samples are in olive and red, respectively. The fitted decay with the 2-k_{FRET} model and the fitting residuals are plotted in black. The fitting residuals with 1-k_{FRET} model are plotted in grey.

Supporting Figure 4

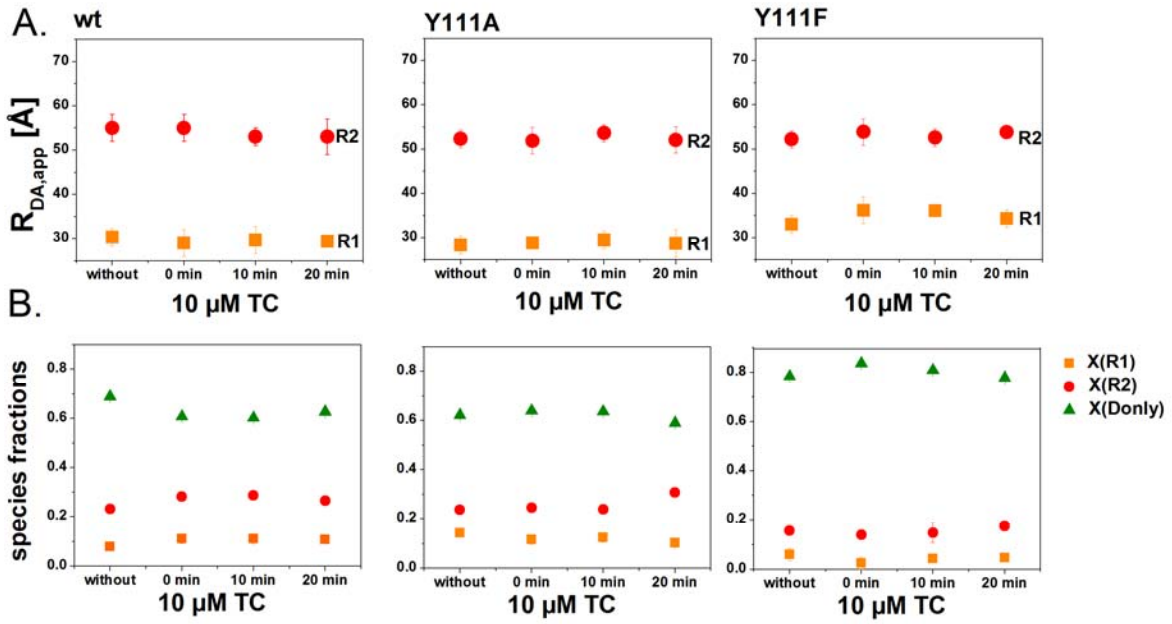

Supporting Figure 4: Time-series analysis after TC ligand stimulation.

HEK293 cells were transiently transfected with TGR5-GFP alone (Donly) or with TGR5-GFP and TGR5-mCherry with the D/A ratio of 1:10. To study changes in FRET after ligand addition, three cells were selected using the Olympus Time laps function and measured at different time points, including before adding 10 µM TC (without), immediately after TC addition (t=0), 10 min after and 20 min after. The apparent distances R_{DA} species fractions were fitted using self-made software. **(a)** The apparent distances are plotted versus time. Each point represents the average of nine cells (three measurements with three cells). **(b)** The species fractions X(R1), X(R2) and the Non-FRET fraction X(Donly) at four time points (representing without TC, t=0, t=10 min and t=20 min) are plotted, but no substantial change due to ligand addition could be detected. Orange=R1=high FRET distance, red= R2= low FRET distance, green=Donly fraction.

Supporting Figure 5

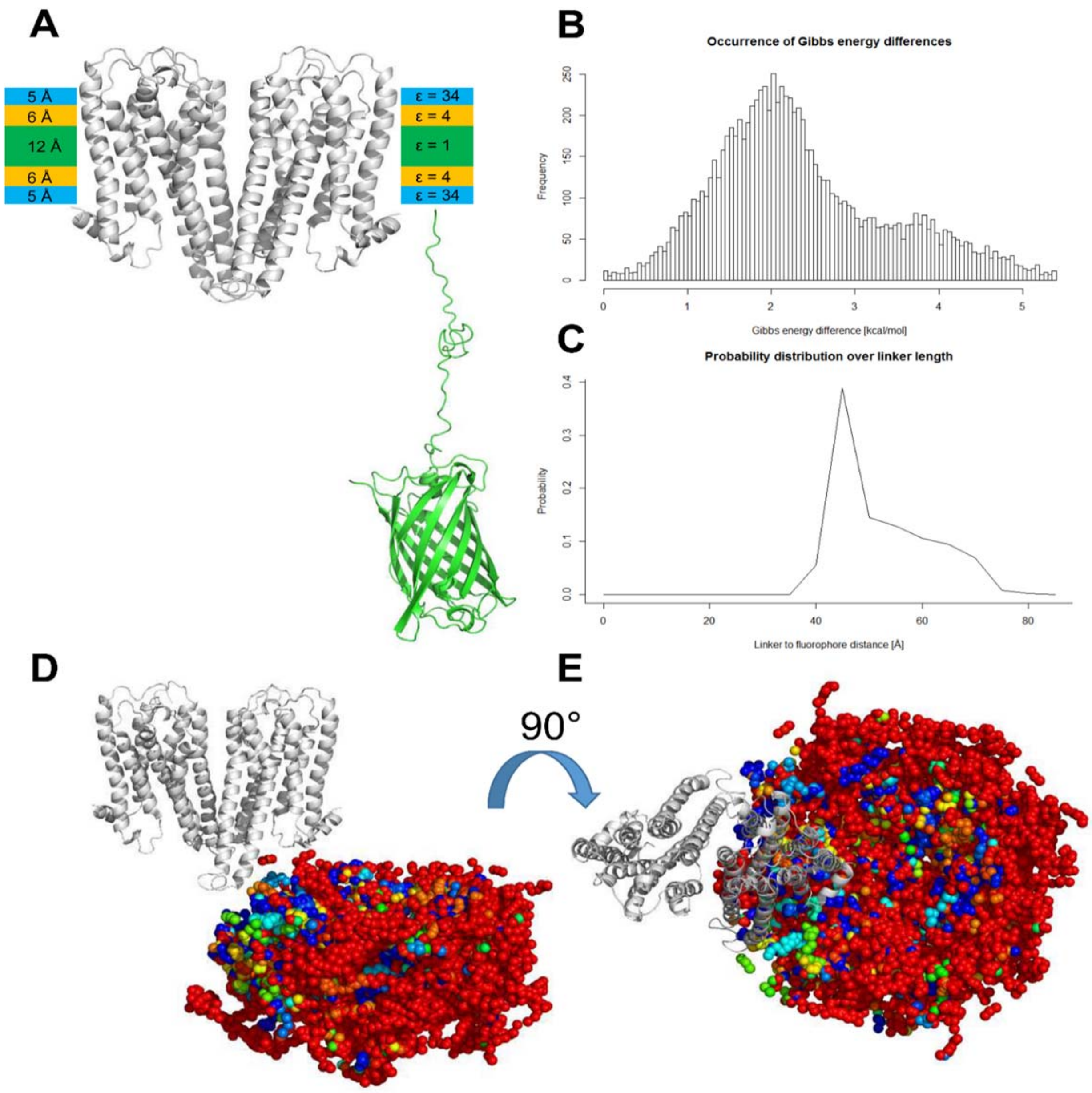

Supporting Figure 5: **Explicit linker/GFP simulation and probability distribution of linker/GFP configurations**.

(a) Starting structure of the TGR5 4/5 dimer (grey) and of the linker and GFP after the initial minimization (green). The linker and GFP were simulated separately from the TGR5 dimer; the structure shown here illustrates one of the composite models used for the MM-PBSA

calculations. At the ‘wad’ in the middle of the linker, several proline residues are present. The positioning of the implicit membrane slabs is shown in colored bars next to the TGR5 dimer. The bars on the left show the thickness of each membrane layer used in the FEW^{mem} calculations, while the bars on the right show the respective electric permittivity. **(b)** Frequency distribution of Gibbs energies (**equation (9)** in the main text) relative to the energetically most favorable snapshot after linear scaling (see main text). **(c)** Probability distribution of the Boltzmann-weighted distance between the fluorophore and the N-terminus of the linker. **(d) + (e)**. Ensemble of linker/GFP configurations represented in terms of the C-alpha atom of the central residue of the fluorophore generated by MD simulations with added rotations in relation to the TGR5 4/5 dimer (grey) in side (**d**) and exoplasmic view (**e**). The coloring of the C-alpha atoms corresponds to their probability ranging from lowest (blue) to highest (red). Conformations with a low probability are more frequently found in close contact to the dimer.

Supporting Figure 6

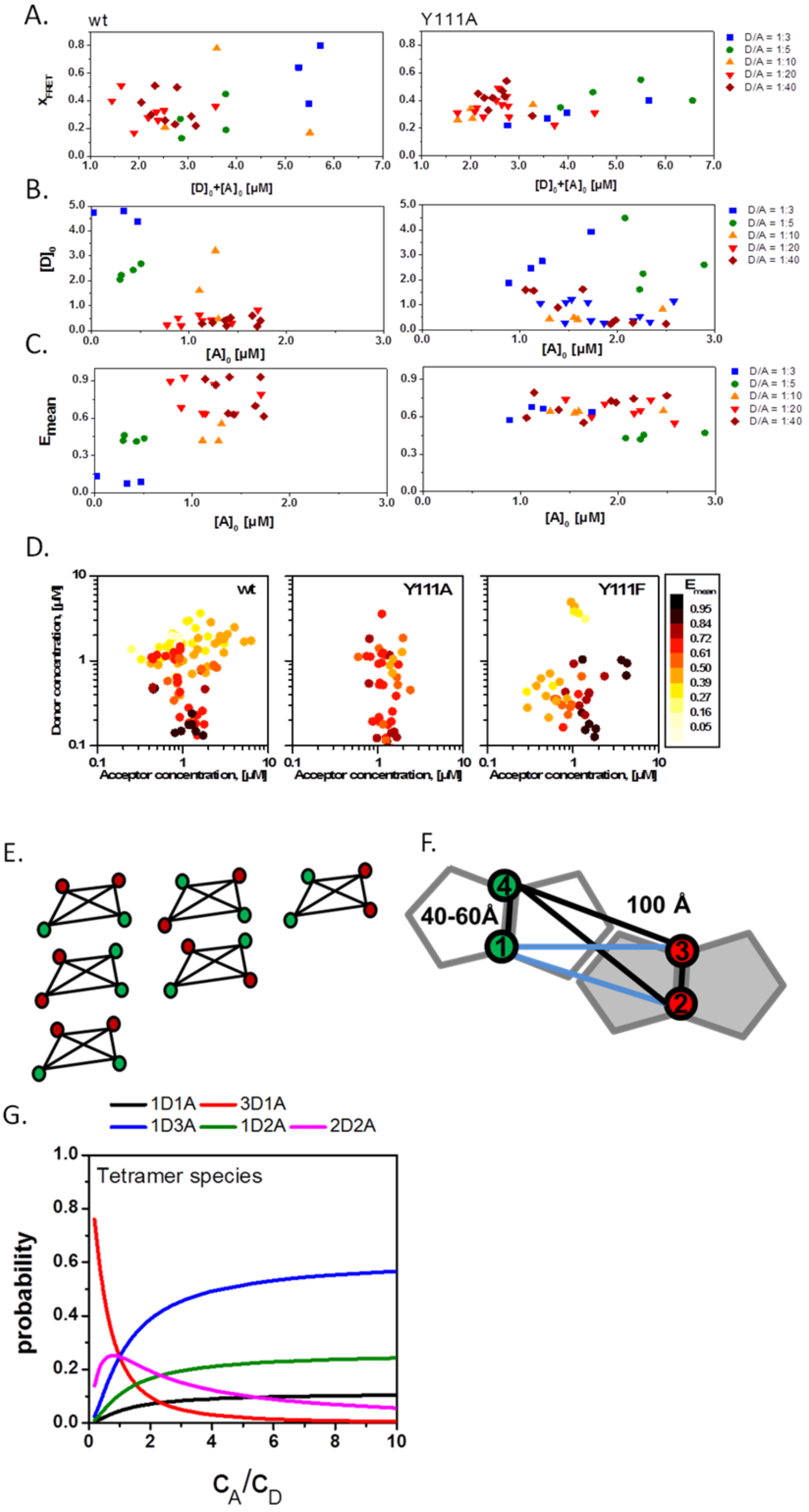

Supporting Figure 6: **Characterization and estimation of the association constants with a dimer/tetramer fit model**.

HEK293 cells were transiently transfected with TGR5 wt (left plots) or Y111A (right plot) donor to acceptor ratios varying from 1:3 to 1:40. **(a)** The total protein concentration $[D]_0$+$[A]_0$ (**eq.(4-6**) and the FRET species fractions x_{FRET} were obtained from MFIS measurements and plotted to calculate the dissociation constant K_D. The FRET species fractions calculated from different D/A ratios were distributed equally in a concentration range of 1-7 μM. From these data K_D cannot be directly determined. The upper limit for K_D should be less than 1 μM. **(b)** The real donor $[D]_0$ and acceptor $[A]_0$ concentrations from the D/A transfection experiments were plotted for wt and Y111A to estimate differences in experimental and real concentration ratios between donor and acceptor. **(c)** E_{mean} increases in an $[A]_0$ dependent manner in wt but not in Y111A transfected cells. **(d)** Overview on the concentration ranges of donor and acceptor and its influence on E_{mean}, whose size is depicted in color. Variant specific interaction patterns are readily visible. **(e)** Description of our data by a minimal dimer/tetramer model to. In this model we assume that a tetramer is constituted of a dimer of dimers. In a tetramer the sum of donor, acceptor and unlabeled molecules is constant (**eqs. (7-9)**). Six tetramer configurations for a case of two acceptor (red) and two donor molecules (green) are possible. **(f)** Composition of a simplified rectangular tetramer molecule with random arrangement of two donors and two acceptors according to a linear organization of the GPCR. The positions of the green and red circles in the pentagram represent the fluorescent proteins attached to helix 8. **(g)** Probabilities of all tetramer species composed of a certain number of donor and acceptors (1D1A, 3D1A, 1D3A, 1D2A, 2D2A) in dependence of the acceptor to donor ratio. In our case the most probable scenario is the 2D2A case which describes our data best.

Supporting Figure 7

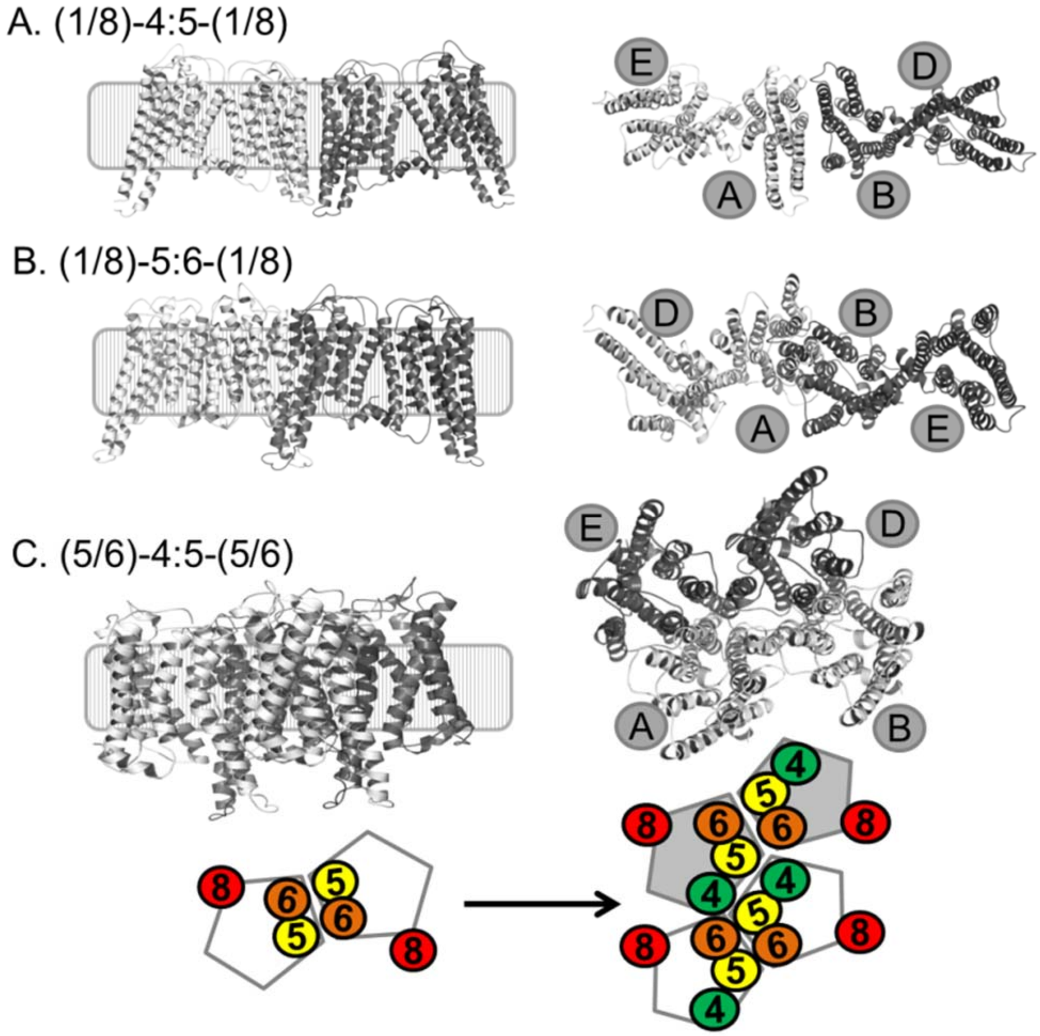

Supporting Figure 7: GPCR tetramer organization and AV simulations.

(a)-(c) Cartoon presentation made with the free software PyMol [1,2] from the membrane view (right) and cytoplasmic view (left) for three possible tetramer organizations. The labels A, B, D and E refer to the TGR5 monomers and are used for distance distribution calculations (**Supporting Table 5**). The corresponding dimers are colored in light grey or dark grey.

Supporting Notes

Proximity FRET

Pixel-wise analysis of the fluorescence data in TGR5 Y111A compared to wt and Y111F showed strong differences in the FRET properties, which were only detectable in an acceptor concentration-dependent manner (**Figure 4,** main text). Thus, we tested whether the observed FRET could simply be caused by a very high local concentration of acceptor proteins in the membrane, so that donor and acceptor are in proximity even though they do not interact. This phenomenon is called "*proximity FRET*".

Due to the single-molecule sensitivity of our confocal microscope, we could perform FRET experiments with acceptor concentrations of ~1-6 µM in 1.23 fl, which corresponds to a molecule density of less than ~0.02 acceptor molecules/nm^2. According to King *et al.*[3], proximity FRET is negligible ($E < 0.1$) at these concentrations.

The pixel-integrated, time-resolved FRET analysis $\varepsilon(t)$ supported the pixel-wise analysis and clearly demonstrated the presence of different FRET species in TGR5 wt and Y111F and therefore the formation of higher-order oligomers as compared to Y111A.

Supporting Methods

1. Molecular biology

Cell culture reagents

Cell culture media were from PAA (Coelbe, Germany). Foetal calf serum (FCS) was from Biochrom (Berlin, Germany). Taurolithocholic acid (TLC), Taurocholic acid (TC) and Forskolin (F) were purchased from Sigma-Aldrich (Taufkirchen, Germany) and Calbiochem (San Diego, CA, USA), respectively.

Cloning of TGR5

Human TGR5 was cloned as previously described [4]. Constructs were cloned into the pcDNA3.1+ (TGR5-pcDNA: complete CDS; TGR5-His: stop codon in complete CDS replaced by C-terminal 8xHis-tag), pGFP-N1, and pmcherry-N1 (stop codon in the complete CDS replaced by a restriction site) vectors. The FLAG-TGR5-YFP construct with an N-terminal FLAG-tag and a C-terminal YFP-tag was cloned into the pEYFP-N1 vector. All vectors were from Clontech, Palo Alto, CA. The Y111A and Y111F mutations were introduced into different TGR5 cDNA constructs using the QuikChange Site-Directed Mutagenesis Kit (Agilent Technologies, Santa Clara, USA) [4]. All cloning strategies and mutagenesis primer sequences can be obtained upon request. Successful cloning and mutagenesis was verified by sequencing (GenBank accession numbers: TGR5:NM_001077191.1).

Immunofluorescence and confocal laser scanning microscopy

Human embryonic kidney 293 (HEK293) cells and Madin Darbin canine kidney cells (MDCK), grown on glass coverslips or transparent filter wells, were transiently transfected with TGR5 wt, Y111A or Y111F in pcDNA3.1+ and pEYFP-N1 vectors using Lipofectamine2000 (Invitrogen)

for 48 h according to the manufacturer's recommendations. After fixation with -20°C cold methanol for 30 sec, cells were incubated with RVLR2 [5] antibody against TGR5 and Cyanine-3 (1:500) conjugated secondary antibodies, which were purchased from Dianova (Hamburg, Germany). Nuclei were stained with Hoechst 34580 (1:20000; Invitrogen). Images were analysed on a Zeiss LSM510META confocal microscope using a multi-tracking modus. A 63 x objective and a scanning resolution of 1024 x 1024 pixels was used for all samples.

Flow cytometry

TGR5 plasma membrane protein amount was quantified by flow cytometry (FACS) using a FACS-CANTO-II (BD Biosciences; Heidelberg, Germany) as previously described [4,6]. HEK293 cells were transiently transfected with the FLAG-TGR5-YFP constructs using Lipofectamine2000. The N-terminal FLAG-tag was detected with the anti-FLAG M2-antibody (Sigma-Aldrich) using the Zenon PacificBlue Label-Kit (Invitrogen) according to manufacturer's instructions. TGR5 plasma membrane expression was calculated by the amount of FLAG-tag positive cells divided by the total amount of TGR5 positive cells as determined by YFP-fluorescence.

Reporter gene assay

HEK293 cells were transiently transfected with TGR5 wt and TGR5 Y111A and TGR5 Y111F variants in the pcDNA3.1+ construct (0.5 ug), pEYFP-N1-empty vector (1.1 ug), reporter PlasmLuc (1.6 ug; Bayer AG; Leverkusen, Germany), and Renilla expression vector (0.1 µg; Promega; Madison, WI, USA) using Lipofectamine2000. The PlasmLuc-reporter gene construct contains 5 cAMP-responsive elements (CREs) upstream of the luciferase gene. Luciferase activity was normalized to transfection efficacy, which was monitored by cotransfection with the Renilla expression vector, and served as measure for the rise in intracellular cAMP. Luciferase

activity was determined 16 hours after stimulation with DMSO, TLC or Forskolin [4,6]. The increase in TLC- and Forskolin-dependent luciferase activity is relative to the DMSO stimulation, which was set equal to 1.0.

Co-immunoprecipitation

HEK293 cells were cotransfected with TGR5-YFP and TGR5-His. Cells transfected with the empty vector (pcDNA or pEYFP-N1) and only one of the TGR5 cDNAs (TGR5-His or TGR5-YFP) served as controls. Cells were lysed with a buffer containing 50 mM Tris-HCl pH 8.0, 1% Nonidet® P40 (AppliChem) and complete protease inhibitor cocktail tablets (Roche). Protein concentration was determined by Bradford assay, and 0.05 mg protein from each sample was set aside as input control. 1.6 mg protein from each sample was used for immunoprecipitation with the μMACS His-tagged protein isolation kit (Miltenyi Biotec, Bergisch-Gladbach, Germany). His-tagged TGR5 was labelled with the anti-His microbeads and loaded onto the MACS columns. His-tagged proteins were eluted from the columns with 60 μl elution buffer and divided into two equal samples of 25 μl each. These as well as the input control samples were subjected to deglycosylation using the N-glycosidase-F Kit (Roche Diagnostics, Mannheim, Germany) for 10 min at 37°C. The deglycosylation reaction was stopped with 10% Laemmli buffer, heated to 95°C for 3 min. IP samples and input controls were separated by SDS page and proteins were transferred to PVDF membranes. His-tagged proteins were detected with the HRP-coupled anti-His antibody (dilution 1:5000, Miltenyi Biotec). YFP-coupled proteins were detected using the HRP-coupled anti-GFP antibody (dilution 1:5000, Miltenyi Biotec). Glyceraldehyde-3-phosphate dehydrogenase (GAPDH) was detected with an antibody from GeneTex (dilution 1:10000) and a secondary HRP-coupled anti-mouse antibody (dilution 1:10000, Dako). Densitometry was performed using the Totallab-100 software (Nonlinear Dynamics, Durham,

NC). The relative amount of TGR5 oligomerization was calculated by dividing the amount of TGR5-YFP protein through the amount of TGR5-His protein. Wildtype TGR5-YFP/TGR5-His was set to 1.0.

2. MFIS-FRET: microscopy and analysis

Sample preparation for MFIS-FRET experiments

For live cell experiments HEK293 were seeded on 8 well chambered glass slides (Labtek, Nunc, USA) one day before transfection. Cells were transiently transfected with 0.5 µg DNA at a density of about 80% using FuGene6 (Promega) according to the manufacturer's protocol 24 to 48 h before analysis. Cell vitality and successful transfection was visually inspected before MFIS measurements.

Microscope calibration

Calibration measurements with Rhodamine 110 delivered the G-factor $G = Sg_{\perp}/Sg_{\parallel}$ for the GFP emission wavelength range (green channels). The G-factor accounts for the detection efficiency difference between detectors of both polarizations ($g_{\perp}$ and $g_{\parallel}$). The instrument response function (IRF) was measured with the back-reflection of the laser beam using a mirror and was used for iterative re-convolution in the fitting process. Furthermore, untransfected cells and water were measured at 488 nm and 559 nm for background determination.

Time series experiments of TGR5 stimulation by Taurocholic acid (TC)

To study the effect of bile acid agonists on the FRET parameters we used the water-soluble ligand TC, because addition of DMSO (necessary to dissolve TLC) affects the fluorescence signal significantly. For the time series experiments the time laps viewer function supplied by Olympus LSM was used. The motorized table was calibrated, and three cells were selected and

monitored over a 40 minutes time period. FRET measurements were taken every 10 minutes: before the addition of TC immediately after addition (t = 0 min), and after ten and twenty minutes (t = 10 min; t = 20 min). Cells were excited with 488 nm and 559 nm laser light as described above. Where necessary, changes in focus and system drift were corrected.

Pixel-wise analysis

To determine fluorescence-weighted lifetimes in a pixel-wise analysis, the histograms presenting the decay of fluorescence intensity after the excitation pulse were built for each pixel with 128 ps per bin. We used a maximum likelihood estimator (MLE) to determine the fluorescence-weighted averaged lifetime of donor molecules $\langle \tau_{D(A)} \rangle_f$ in a single pixel using a model function containing only two variables, $\langle \tau_{D(A)} \rangle_f$ and the scatter contribution fraction.

MFIS-FRET 2D histograms

For oligomerization analysis, we plotted the 2D histograms of donor lifetime $\langle \tau_{D(A)} \rangle_f$ vs the green to yellow fluorescence intensity ratio (F_D/F_A) (see equations (2) and (3)) corrected for crosstalk (characterized by the crosstalk factor α), background $\langle B \rangle$, detection efficiencies of D (g_G) and A (g_Y). The acceptor fluorescence used for 2D-FRET must also be corrected for additional direct acceptor excitation DE and relative concentration dependent brightness DE_{rel}. Furthermore all samples were corrected for distinct fluorescence quantum yields Φ and a spectral shift factor γ (especially for TGR5 Y111A) which is considered in the corrected green detection efficiency (g_G*).

$$F_D = \frac{S_G - \langle B_G \rangle}{g_G *} \quad (1)$$

$$F_A = \frac{S_Y - (\langle B_Y \rangle + DE_{rel}) - \alpha(S_G - \langle B_G \rangle)}{g_Y} \quad (2)$$

The crosstalk factor α is determined as the ratio between donor photons detected in the yellow channels and those detected in the green channels for the Donor only (Donly) labeled sample. The corrected detection efficiency g_G* is determined as the ratio of the spectral shift influenced by green detection (0.69) and expected green detection (1.12) multiplied with the quantum yield Φ_{Y111A} obtained from a self-made detection efficiency software. The F_D/F_A parameters for each variant are provided in **Supporting Table 1**

The simultaneous reduction in both FRET indicators $\langle \tau_{D(A)} \rangle_f$ and (F_D/F_A) indicate FRET due to proteins interaction. For a given sub-population selection of the donor fluorescence decay histogram with 32 ps time resolution was constructed for further pixel-integrated sub-ensemble analysis, and the species-averaged fluorescence lifetime of the donor $\langle \tau_{D(A)} \rangle_x$ was calculated based on fit results (species fractions x_i and lifetimes $\tau_{D(A)}$)

$$\langle \tau_{D(A)} \rangle_x = \sum_{i=1}^{n} x_i \cdot \tau_{D(A),i} \quad (3)$$

n is the number of exponents used in donor fluorescence lifetime fitting.

Determination of acceptor and donor concentration from MFIS experiments

TGR5 monomers were either labelled with donor or acceptor fluorescent proteins and transiently transfected into cells with different donor-to-acceptor concentration ratios. The fractions of active donor (denoted as D) and active acceptor (denoted as A) is f_D and f_A respectively. The rest are inactive FPs, which we considered as dark (i.e. no fluorescence emission) and dysfunctional (i.e. FRET-negative). To calculate the protein concentrations from fluorescence intensity, the detection volume of our microscope and GFP and mCherry brightness are required. The

detection volume was determined as $1.23*10^{-15}$ l from FCS measurements of Cyanine 3B (Cy3B). The fitting model applied to the obtained FCS curve assumes a 3-dimensional Gaussian-shaped volume, and a single diffusing species including transitions to a triplet state as described in [7]. The brightness of enhanced GFP and mCherry *in vivo* were individually characterized from FCS measurements of freely diffusing FPs in cytoplasm. We found that with 0.6 μW of 559 nm laser excitation at the objective, mCherry brightness is 0.68 kcpm in cytoplasm. With 0.4 μW of 485 nm laser excitation at the objective, GFP brightness is 0.56 kcpm in cytoplasm.

The average mCherry fluorescence intensity of an image with mCherry excitation ($S_{Y,Y}$) was first corrected for detector dead time, and then used to calculate the total concentration of mCherry, $[A]_0$, with the determined detection volume and the mCherry brightness:

$$[A]_0 = \frac{S^m_{Y,Y}}{brightness\ [kcpm] * confocal\ volume\ [fl]} \tag{4}$$

$$= \frac{S^m_{Y,Y}}{0.68\ kcpm * 0.8\ fl}$$

The average GFP fluorescence intensity of an image with GFP excitation was also corrected for detector dead time, and then the obtained intensity ($S^m_{G,G}$) was further corrected for the quenching effect due to FRET:

$$S_{G,G} = \frac{S^m_{G,G}}{(1 - x_{FRET}) + x_{FRET} \cdot (1 - E)} \tag{5}$$

$S_{G,G}$ is the unquenched GFP fluorescence intensity in the absence of FRET, the energy transfer efficiency E and fraction of FRET-active population, x_{FRET}, were calculated as described in the main method sections. $S_{G,G}$ was then used to calculate the total concentration of GFP, $[D]_0$. The wavelength dependent confocal volume is 0.5 fl.

Assuming the concentration of the FPs reflects the concentration of their host proteins, the TGR5 concentration (without non-fluorescent molecules) in μM was determined as:

$$\text{protein concentration} = [A]_0 + [D]_0 = c_A + c_D \tag{6}$$

Estimation of the association constants for oligomerization

The total protein concentration and the protein association constants have to be considered to determine the oligomerization state or the chemical speciation. To calculate the transfer-efficiency for a given oligomerization the spatial organization of the molecules within the oligomers and the concentration of donor, acceptor and non-fluorescent molecules has to be considered. The total protein concentration (**equation (6)**) is given by the sum of the acceptor, the donor and the unlabeled protein concentration:

$$c_T = c_A + c_D + c_U \tag{7}$$

Here the unlabeled protein concentration c_u equals the concentration of immature mCherry. The protein concentrations were calculated using the brightness of free GFP and free mCherry as reference Even though higher-order oligomerization is anticipated we used a simple dimer/tetramer model to describe our data as this allows for a quantitative description. In this model we assume that a tetramer is constituted of a dimer of dimers (**Supporting Fig. 6**). Hence, starting from a monomer two equilibriums have to be treated:

$$\begin{aligned} o + o \quad &\rightarrow \quad oo \quad K_1 = \frac{[oo]}{[o][o]} \\ oo + oo \quad &\rightarrow \quad (oo)(oo) \quad K_2 = \frac{[(oo)(oo)]}{[oo][oo]} \end{aligned} \tag{8}$$

Here o is a monomer, oo a dimer and (oo)(oo) is a tetramer. We use the monomer o as a master species. Then the total protein concentration is given by:

$$c_T = [o] + 2 \cdot [oo] + 4 \cdot [(oo)(oo)] \tag{9}$$

Now, the concentrations of the three species o, oo and (oo)(oo) for any given the total protein concentration is obtained by solving the three equations above.

To calculate the transfer efficiency we assume that donor, acceptor and unlabeled molecules behave biochemically identical. Hence, the probability of an oligomer composition is given by the probability of finding a donor, acceptor or unlabeled molecule and the counting statistics. The probabilities of finding a donor, acceptor or unlabeled molecule depend on their respective concentrations. For instance the probability of an acceptor molecule is given by the respective species and total protein concentration:

$$p_A = \frac{c_A}{c_T} \tag{10}$$

In a tetramer the sum of donor, acceptor and unlabeled molecules is constant. The probability of a certain tetramer composition is obtained by the multinomial distribution:

$$\begin{aligned} p(n_D, n_A, n_U) &= N \cdot p_D^{n_D} p_A^{n_A} p_U^{n_U} \\ &= \frac{(n_D + n_A + n_U)!}{n_D! n_A! n_U!} \cdot p_D^{n_D} p_A^{n_A} p_U^{n_U} \end{aligned} \tag{11}$$

N is the number of combinations for a given composition. Each combination might have a different FRET-rate distribution. Hence, in case of two donors and two acceptors 6 combinations as shown in **Supporting Fig. 6** contribute to the signal. If only species with at least one donor and one acceptor are considered the FRET-rate constants of overall 38 distinct species and their respective probabilities and FRET-rate constant distributions have to be calculated. The species

probabilities summarized by their donor and acceptor composition in dependence of the acceptor to donor ratio c_A/c_D are illustrated in **Supporting Fig. 6**.

FRET-rate constants are additive. Therefore in case of multiple acceptors the total FRET-rate constant experienced by a donor (i) is given by the sum of all FRET-rate constants of all acceptors (j):

$$k_{RET}^{(i)} = \frac{1}{\tau_0} \cdot \sum_j \left(\frac{R_{DA}^{(ij)}}{R_0} \right)^6 \tag{12}$$

Here $R_{DA}^{(ij)}$ is the donor acceptor distance between the donor (i) and the acceptor (j) which is determined by the spatial arrangement of the oligomer. For instance, in the case as illustrated in **Supporting Fig. 6** the two FRET-rates experienced by the donor at position 1 and the donor at position 4 are given by:

$$k_{RET}^{(1)} = \frac{1}{\tau_0} \cdot \left(\left(\frac{R_{DA}^{(13)}}{R_0} \right)^6 + \left(\frac{R_{DA}^{(12)}}{R_0} \right)^6 \right) \tag{13}$$

$$k_{RET}^{(4)} = \frac{1}{\tau_0} \cdot \left(\left(\frac{R_{DA}^{(42)}}{R_0} \right)^6 + \left(\frac{R_{DA}^{(43)}}{R_0} \right)^6 \right)$$

These FRET-rates result in first approximation in bi-exponential fluorescence decay, if the coupling between the two donors is not considered.

For a given structural arrangement all FRET-rate constants for all possible compositions (one donor one acceptor, two donors one acceptor, etc.) were calculated (**Supporting Fig. 6**). Later the average transfer-efficiencies of the tetramer compositions containing at least one donor and one acceptor were calculated.

It has to be considered that the contribution to the fluorescence signal depends on the number of donor molecules. For instance a tetramer constituted out of three donors and one acceptor molecule contributes three times more to the total signal as compared to a tetramer only constituted out of one donor, one acceptor and two unlabeled molecules.

The predicted transfer efficiency for each data point depends now only on the equilibrium association constants K_1, K_2 and the spatial arrangement of the fluorophores in the dimer and the tetramer. To reduce the number of free parameters we assumed that the tetramer can be described by a rectangular geometry where one edge is approximately 100 Å long while the second edge is between 40-50 Å long (**Supporting Fig**. **6**). This assumption is in line with the homology models (**Supporting Table 5** and **Supporting Fig**. **7**). Furthermore, only FRET molecules have been selected. Therefore, the first equilibrium from monomer to dimer is not monitored and only the equilibrium constant of the tetramer formation is probed. Thus, only K_2 and the dimer distance in the range of 40-60 Å is reflected in the data. For the measurements we find that a short distance of approximately 45 Å describes the data best. For the TGR5 wt and Y111F variant we find predominately a tetrameric or higher-order oligomer configuration while in case of the Y111A mutant the molecules are predominately in a dimeric configuration.

Statistical analysis

Experiments were performed independently at least three times. For MFIS-FRET at least nine cells per series in three independent experiments were measured. Results are expressed as mean ± standard error of the mean (SEM) and analysed using the two-sided student t-test. A $p \leq 0.01$ was considered statistically significant.

3. Molecular modelling and simulation

Structural models of TGR5 dimers and tetramers

Dimer models with the interface TM1 and H8 (1/8) were generated by structurally aligning two homology models of TGR5 [8] onto the dimeric crystal structure of the human κ-opioid receptor (PDB ID: 4DJH [9]) via the 'cealign' command in Pymol [2]. For dimer models with the 4/5 interface and the 5/6 interface the same procedure was applied using the human CXCR4 receptor (PDB ID: 3ODU [10]) and the murine μ-opioid receptor (PDB ID: 4DKL [11]) as alignment templates, respectively.

Tetramer models were built in a similar fashion. Here, two TGR5 dimers with the same dimer interface, e.g. (1/8), were aligned on another TGR5 dimer with a different interface, e.g. (4/5). With this procedure six tetramers were generated: (1/8) and (5/6) dimers with an oligomeric interface of (4/5); (1/8) and (4/5) dimers with an oligomeric interface of (5/6); (4/5) and (5/6) dimers with an oligomeric interface of (1/8). Subsequently, the interface residues of the respective dimer and tetramer models were energy minimized in Maestro [12,13] using the VSGB 2.0 solvation model [14]. Finally, either dimer and tetramer model were submitted to the OPM server [15] to compute its orientation in a membrane.

Explicit linker simulations: Molecular dynamics simulations of GFP bound to a linker

For computing a thermodynamic ensemble (TE) of GFP positions with an explicit linker/GFP construct, initially, the structure of the TGR5 C-terminal residues 296-330, for which no experimental structural information is available, and the nine residues that connect the C-terminus to GFP (total sequence: QRCLQGLWGRASRDS PGPSIAYHPSSQSSVDLDLN YGSTGRHVS) was generated with the 'Protein building' approach in Maestro. Phi and psi angles of zero were chosen, resulting in a straight peptide conformation and, hence, a structurally

unbiased starting structure for the molecular dynamics (MD) simulations. This linker was subsequently fused to enhanced GFP (PDB ID: 4EUL [16]), and the resulting structure was capped with acetyl and *N*-methyl amide groups at the N- and C-termini, respectively, and protonated with PROPKA[17] according to pH 7.4. We assumed the thermodynamic ensemble (TE) of mCherry to be identical to that of GFP.

Then, the linker/GFP construct was neutralized by adding counter ions and solvated in an octahedral box of TIP3P water [18] with a minimal water shell of 12 Å around the solute. The Amber14 package of molecular simulation software [19,20] and the ff14SB and GAFF [21] force fields were used to perform an all-atom MD simulations. To cope with long-range interactions, the "Particle Mesh Ewald" method [22] was used, and the SHAKE algorithm [23] was applied to bonds involving hydrogen atoms. The time step for all MD simulations was 2 fs with a direct-space, non-bonded cut-off of 8 Å. The first linker residue was fixed with positional harmonic restraints with a force constant of 100 kcal mol^{-1} $Å^{-2}$ throughout the simulations to emulate that this residue would be bound to TGR5 embedded in a membrane. At the beginning, 17500 steps of steepest decent and conjugate gradient minimization were performed; during 2500, 10000, and 5000 steps positional harmonic restraints with force constants of 25 kcal mol^{-1} $Å^{-2}$, 5 kcal mol^{-1} $Å^{-2}$, and zero, respectively, were applied to the solute atoms. Thereafter, 50 ps of NVT-MD (MD simulations with a constant number of particles, volume, and temperature) were conducted to heat up the system to 100 K, followed by 300 ps of NPT-MD (MD simulations with a constant number of particles, pressure, and temperature) to adjust the density of the simulation box to a pressure of 1 atm and to heat the system to 300 K. During these steps, a harmonic potential with a force constant of 10 kcal mol^{-1} $Å^{-2}$ was applied to the solute atoms. As the final step in thermalization, 300 ps of NVT-MD simulations were performed while gradually reducing the restraint forces on the solute atoms to zero within the first 100 ps of this step. Afterwards, six

independent production runs of NVT-MD simulations with 150 ns length each were performed. For this, the starting temperatures of the simulations at the beginning of the thermalization were varied by a fraction of a Kelvin. The conformations obtained in these simulations were pooled for further analyses.

Implicit linker simulations

Inter-dye distance distributions for all TGR5 dimer and tetramer models were calculated using an modified Accessible Volume (AV) approach [24]. Firstly, the different protein models (see 5.14) were embedded in an explicit membrane via the CHARMM-GUI membrane builder [25]. Here, a membrane with 5500 lipids of 1,2-dioleoyl-*sn*-glycero-3-phosphocholine (DOPC) per layer was created employing default settings of the CHARMM-GUI. This resulted in a membrane bilayer of about 1.5 million atoms and a side length of about 620 Å to prevent the linker/GFP construct (which has an extended length of about ~229 Å) from wrapping around the edge of the membrane. As neither ions nor water were needed for AV calculations, the steps of ion and water addition were omitted during the creation of the membrane.

For the AV simulations the fluorescent probe was attached to the C-terminal amino acid of the TGR5 via a flexible linker of 203.5 Å corresponding to 55 amino acids (36 amino acids of the TGR5 C-terminus, a 6 amino acid cloning linker, and the first 13 amino acids of the GFP's (PDB ID: 4EUL) N-terminus, **see Supporting Table 2**) with a length of 3.7 Å each [26]. A dye radius of 25 Å was used as an approximation for the GFP size, resulting in a total length of ~229 Å for the linker/GFP construct. The distance between linker attachment points in most of the screened oligomer models was shorter than the effective size of the AVs resulting in AV overlap. The AVs were constructed considering geometric factors in terms of steric exclusion effects caused by the TGR5 oligomer and the membrane. To account for clashes between the dyes, which are

not addressed in the AV simulations, the inter-dye distance probability was set to zero for all distances below 25 Å. To account also for entropic effects, we introduced position weights for the implicitly modelled linker according to the Gaussian chain model, so that the non-uniform dye position probability distribution in the AV was scaled (**Supporting Fig. 7**) [27]. In the Gaussian chain model a segment length of 7.4 Å was used, as obtained from the calibration aimed to reproduce the accurate end-to-end distance probability distribution from coarse-grained Monte-Carlo simulations of the peptide linker, similar to previously published results for the flexibly linked GFP dimer [28]. The obtained AV positional distributions were used to determine the inter-probe distance distribution by measuring all distances from positions in one AV distribution with respect to all positions in the second AV distribution. Considering oligomerization (tetramer) where two acceptors may be present in the vicinity of one donor, we computed the apparent distance distribution shifts towards shorter distances by convolution of the two inter-probe distance distributions ((1/8) and (4/5)) (**Supporting Fig. 5**).

Glossary

F_D/F_A	fluorescence intensity ratio
B	background
α	crosstalk factor
g	detection efficiencies
DE	direct acceptor excitation
DE_{rel}	relative concentration dependent brightness
Φ	distinct fluorescence quantum yields
γ	spectral shift factor
g_G*	corrected green detection efficiency
$\varepsilon(t)$	pixel-integrated, time-resolved FRET analysis
DA	donor acceptor FRET pair
$f(t)$	fluorescence signal decay
D(0)	unquenched donor
D(A)	Donor quenched by acceptor
IRF	instrument response function
x<br>xD<br>xA	species fraction<br>donor (Donly) or Non-FRET fraction<br>acceptor fraction
AV	Accessible Volume
$\langle R_{DA}\rangle_{app}$	Apparent mean distance between Donor and Acceptor
$\langle \tau_{D(0)}\rangle_f$	Fluorescence-averaged unquenched donor fluorescence lifetime
$S_{em,ex}$<br>$S_{G,G}$<br>$S_{Y,G}$	$\text{Signal}_{\text{emission, excitation}}$<br>Signal of green photons emitted after excitation of GFP<br>Signal of mCherry photons emitted after excitation of GFP
c<br>$[D]_0, [A]_0$	Concentration<br>Real donor and acceptor concentration

E_{mean}	Mean Transfer efficiency
wt	wildtype
MFIS-FRET	Multiparameter Fluorescence Imaging Spectroscopy-Förster Resonance Energy Transfer

Supporting References

1 DeLano, W. L. & Lam, J. W. PyMOL: A communications tool for computational models. *Abstr Pap Am Chem S* **230**, U1371-U1372 (2005).

2 Schrödinger, L. The PyMOL molecular graphics system, version 1.3 r1. Py-MOL, The PyMOL Molecular Graphics System, Version 2010,. (2010).

3 King, C., Sarabipour, S., Byrne, P., Leahy, D. J. & Hristova, K. The FRET Signatures of Noninteracting Proteins in Membranes: Simulations and Experiments. *Biophys J* **106**, 1309-1317, doi:10.1016/j.bpj.2014.01.039 (2014).

4 Spomer, L. *et al.* A Membrane-proximal, C-terminal alpha-Helix Is Required for Plasma Membrane Localization and Function of the G Protein-coupled Receptor (GPCR) TGR5. *J Biol Chem* **289**, 3689-3702, doi:10.1074/jbc.M113.502344 (2014).

5 Keitel, V. *et al.* The Membrane-Bound Bile Acid Receptor TGR5 Is Localized in the Epithelium of Human Gallbladders. *Hepatology* **50**, 861-870, doi:10.1002/hep.23032 (2009).

6 Hov, J. R. *et al.* Mutational Characterization of the Bile Acid Receptor TGR5 in Primary Sclerosing Cholangitis. *Plos One* **5**, doi:e1240310.1371/journal.pone.0012403 (2010).

7 Weidtkamp-Peters, S. *et al.* Multiparameter fluorescence image spectroscopy to study molecular interactions. *Photochem Photobiol Sci* **8**, 470-480, doi:10.1039/b903245m (2009).

8 Gertzen, C. G. W. *et al.* Mutational mapping of the transmembrane binding site of the G-Protein coupled receptor TGR5 and binding mode prediction of TGR5 agonists. *Eur J Med Chem* doi:10.1016/j.ejmech.2015.09.024 (2015).

9 Wu, H. *et al.* Structure of the human kappa-opioid receptor in complex with JDTic. *Nature* **485**, 327-U369, doi:10.1038/nature10939 (2012).

10 Wu, B. *et al.* Structures of the CXCR4 Chemokine GPCR with Small-Molecule and Cyclic Peptide Antagonists. *Science* **330**, 1066-1071, doi:10.1126/science.1194396 (2010).

11 Manglik, A. *et al.* Crystal structure of the mu-opioid receptor bound to a morphinan antagonist. *Nature* **485**, 321-U170, doi:10.1038/nature10954 (2012).

12 Maestro (Schrödinger, LLC., New York, NY, 2014).

13 Version 9.9.013 (Schrödinger LLC, New York, NY, 2014).

14 Li, J. *et al.* The VSGB 2.0 model: A next generation energy model for high resolution protein structure modeling. *Proteins-Structure Function and Bioinformatics* **79**, 2794-2812, doi:10.1002/prot.23106 (2011).

15 Lomize, M. A., Lomize, A. L., Pogozheva, I. D. & Mosberg, H. I. OPM: Orientations of proteins in membranes database. *Bioinformatics* **22**, 623-625, doi:10.1093/bioinformatics/btk023 (2006).

16 Arpino, J. A. J., Rizkallah, P. J. & Jones, D. D. Crystal Structure of Enhanced Green Fluorescent Protein to 1.35 angstrom Resolution Reveals Alternative Conformations for Glu222. *Plos One* **7**, doi:e4713210.1371/journal.pone.0047132 (2012).

17 Bas, D. C., Rogers, D. M. & Jensen, J. H. Very fast prediction and rationalization of pK(a) values for protein-ligand complexes. *Proteins-Structure Function and Bioinformatics* **73**, 765-783, doi:10.1002/prot.22102 (2008).

18 Jorgensen W, C. J., Madura JD, Impey R and Klein ML. Comparison of simple potential functions for simulating liquid water. *The Journal of Chemical Physics* **79**, doi:10.1063/1.445869 (1983).

19 Case, D. A. *et al.* The Amber biomolecular simulation programs. *J Comput Chem* **26**, 1668-1688, doi:10.1002/jcc.20290 (2005).

20 D.A. Case, V. B., J.T. Berryman, R.M. Betz, Q. Cai, D.S. Cerutti, T.E. Cheatham, III, T.A. Darden, R.E. Duke, H. Gohlke, A.W. Goetz, S. Gusarov, N. Homeyer, P. Janowski, J. Kaus, I. Kolossváry, A. Kovalenko, T.S. Lee, S. LeGrand, T. Luchko, R. Luo, B. Madej, K.M. Merz, F.

Paesani, D.R. Roe, A. Roitberg, C. Sagui, R. Salomon-Ferrer, G. Seabra, C.L. Simmerling, W. Smith, J. Swails, R.C. Walker, J. Wang, R.M. Wolf, X. Wu and P.A. Kollman AMBER 14. (2014).

21 Wang, J. M., Wolf, R. M., Caldwell, J. W., Kollman, P. A. & Case, D. A. Development and testing of a general amber force field. *J Comput Chem* **25**, 1157-1174, doi:10.1002/jcc.20035 (2004).

22 Darden, T., York, D. & Pedersen, L. PARTICLE MESH EWALD - AN N.LOG(N) METHOD FOR EWALD SUMS IN LARGE SYSTEMS. *J Chem Phys* **98**, 10089-10092, doi:10.1063/1.464397 (1993).

23 Ryckaert, J. P., Ciccotti, G. & Berendsen, H. J. C. NUMERICAL-INTEGRATION OF CARTESIAN EQUATIONS OF MOTION OF A SYSTEM WITH CONSTRAINTS - MOLECULAR-DYNAMICS OF N-ALKANES. *J Comput Phys* **23**, 327-341, doi:10.1016/0021-9991(77)90098-5 (1977).

24 Kalinin, S. *et al.* A toolkit and benchmark study for FRET-restrained high-precision structural modeling. *Nat Methods* **9**, 1218-1225, doi:10.1038/nmeth.2222 (2012).

25 Jo, S., Lim, J. B., Klauda, J. B. & Im, W. CHARMM-GUI Membrane Builder for mixed bilayers and its application to yeast membranes. *Biophys J* **97**, 50-58, doi:10.1016/j.bpj.2009.04.013 (2009).

26 Evers, T. H., van Dongen, E. M., Faesen, A. C., Meijer, E. W. & Merkx, M. Quantitative understanding of the energy transfer between fluorescent proteins connected via flexible peptide linkers. *Biochemistry* **45**, 13183-13192, doi:10.1021/bi061288t (2006).

27 Chiang, J., Li, I., Pham, E. & Truong, K. FPMOD: a modeling tool for sampling the conformational space of fusion proteins. *Conference proceedings : ... Annual International Conference of the IEEE Engineering in Medicine and Biology Society. IEEE Engineering in Medicine and Biology Society. Conference* **1**, 4111-4114, doi:10.1109/IEMBS.2006.259224 (2006).

28 Evers, T. H., van Dongen, E. M., Faesen, A. C., Meijer, E. W. & Merkx, M. Quantitative understanding of the energy transfer between fluorescent proteins connected via flexible peptide linkers. *Biochemistry* **45**, doi:10.1021/bi061288t (2006).
